# SNORD15B and SNORA5C: Novel Diagnostic and Prognostic Biomarkers for Colorectal Cancer

**DOI:** 10.1155/2022/8260800

**Published:** 2022-05-09

**Authors:** Liping Shen, Wenqing Lu, Yujv Huang, Junyan He, Qi Wang, Xiaofei Zheng, Zhidong Wang

**Affiliations:** ^1^Department of Radiobiology, Beijing Key Laboratory for Radiobiology, Beijing Institute of Radiation Medicine, Beijing 100850, China; ^2^College of Life Sciences, Hebei University, Baoding, Hebei 071002, China; ^3^The First Affiliated Hospital, Department of Radiation Oncology, Hengyang Medical School, University of South China, Hengyang, Hunan 421001, China

## Abstract

Colorectal cancer (CRC) is presenting a global public health problem with high incidence and mortality. Early diagnosis and treatment are the most important strategies to improve prognosis of this disease. Besides fecal occult blood test (FOBT) and colonoscopy, the most widely used methods for CRC screening currently, more effective methods for early diagnosis or prognostic prediction for CRC are needed. Small nucleolar RNAs (snoRNAs) is a class of noncoding RNAs (ncRNAs) playing crucial roles in carcinogenesis and considered to be promising tumor biomarker. In this study, we found that SNORD15B, SNORD48, and SNORA5C were significantly upregulated in CRC tissues. High levels of SNORD15B, SNORD48, or SNORA5C predicted poor clinical outcomes of CRC patients. Forced expression of SNORD15B or SNORA5C in CRC cells promoted proliferation and colony formation. In a further investigation, association between the level of SNORD15B/SNORA5C and clinicopathological parameters of CRC patient cohorts was analyzed based on data from The Cancer Genome Atlas (TCGA). We found that high expressions of SNORD15B and SNORA5C were significantly associated with age, lymphatic invasion, and history of colon polyps, and they were proved to be independent risk factors for survival of CRC patients. This study confirms that SNORD15B and SNORA5C have oncogenic effects in carcinogenesis of CRC. The findings suggest the two genes as potential diagnostic and prognostic biomarkers for CRC.

## 1. Introduction

Colorectal cancer (CRC) is a common malignant tumor presenting as a serious public health problem in worldwide [1]. According to current research, incidence of CRC increases significantly after the age of 40, peaking at the age of 75-79 [2]. The incidence in developed country, urban, and eastern regions of China is higher when compared to developing country, rural areas, and western regions of China [3–5]. According to the 2020 global cancer data from WHO, there were 1.94 million new CRC cases worldwide and 940,000 deaths of them, accounting for both the second highest incidence rate and mortality rate. Epidemiologic study shows that tumorigenesis of CRC is affected by various factors, e.g., heredity, age, lifestyle, environment, gut microbiome, and chronic inflammation [5, 6]. In the early stage of CRC, no obvious clinical symptoms could be observed, yet presentations such as changes in bowel habits, blood in stool, diarrhoea, abdominal pain, anaemia, and weight loss would occur as disease progressing to advanced stages. Diagnosis of CRC in early stage is a key step to eliminate the tumor foci to prolong survival [1, 5]. Currently, fecal occult blood test (FOBT) and colonoscopy remain the most widely used methods for early CRC screening [5]. But both methods have limitations—FOBT is inaccurate in some instances, and colonoscopy is performed entirely by clinicians. Thus, better substitute technologies for early diagnosis or prognostic prediction of CRC are needed, e.g., effective biomarkers.

Small nucleolar RNAs (snoRNAs) are a class of noncoding RNAs (ncRNAs) about 60-300 nucleotides in length and predominantly located in nucleoli. In vertebrates, snoRNAs are generally derived from alternative splicing of intron regions in host genes. They are primarily divided into two categories: C/D box snoRNA and H/ACA box snoRNA. The main function of snoRNAs is to direct posttranscriptional modification of targeted ribosomal RNAs (rRNAs) or small nuclear RNAs (snRNAs). C/D box snoRNAs are generally involved in 2′-O-methylation, while H/ACA box snoRNAs are mainly involved in pseudouridylation. Emerging evidences indicate that snoRNAs can regulate tumor disease processes at multiple levels but not just act as unglamorous “house-keeping genes” as once thought. As more and more evidences accumulated, snoRNAs show promising prospects in tumor diagnosis, diagnostic prediction, and targeting therapy, which makes these transcripts attract much attentions in the field of oncology. Researchers have found that some snoRNAs have been aberrantly expressed in multiple malignant tumors and other diseases [7, 8]. Recent studies indicate that snoRNAs have vital roles in the progression of CRC. SNORA21 and SNORA42 are negatively correlated with the survival rate of CRC patients; overexpression of them promotes proliferation, migration, and invasion of CRC cells and also enhances tumorigenicity [9, 10]. Thus, studies on biological function of snoRNAs are expected to provide novel biomarkers for CRC.

In the present study, we identified SNORD15B and SNORA5C which were involved in CRC carcinogenesis. We assessed differential expression of the two genes in CRC tissues and adjacent normal tissues and validated their biological effects on proliferation and colony formation in CRC cells. Based on clinical patient cohort analysis and in vitro experiments, our data suggested that SNORD15B and SNORA5C are novel oncogenes in the pathogenesis of CRC and could serve as potential biomarkers for diagnosis and prognostic prediction.

## 2. Methods and Materials

### 2.1. Patients and Samples

Fresh cold-frozen CRC tumor tissue and adjacent normal mucosa tissue specimens from 41 patients were analyzed in our study. Tissues were obtained from Liaoning Cancer Hospital (China). The protocol was approved by the Ethics Committee on Human Investigation of the Liaoning Cancer Hospital, and the written informed consent was obtained from all patients. Detailed information of patients' demographics and clinicopathological characteristics was provided in supplementary materials Table S1.

### 2.2. Cell Culture

HCT116, SW620, and HT29 cells were purchased from GeneChem (China) and FHC cells were obtained from ATCC (USA). HCT116 and SW620 cells were cultured in RPMI-1640 medium and HT29 cells were cultured in Dulbecco's Modified Eagle Medium (HyClone, USA). Both of the mediums were supplemented with 10% fetal bovine serum (ExCell Bio, China) and 1% penicillin-streptomycin (HyClone, USA). FHC was cultured in DMEM: F-12 (ATCC, USA), supplemented with 10 mM HEPES (at a final concentration of 25 mM), 10 ng/mL cholera toxin, 0.005 mg/mL insulin, 0.005 mg/mL transferrin, 100 ng/mL hydrocortisone, 20 ng/mL human recombinant EGF (Thermo Fisher PHG0311), 10% fetal bovine serum (GIBCO), and 1% penicillin-streptomycin. All cells were incubated at 37°C in a humidified atmosphere containing 5% CO2.

### 2.3. RNA Extraction and Quantitative Real-Time Polymerase Chain Reaction (qRT-PCR)

Clinical tissues were homogenized in 1 mL TRIzol reagent (Sigma, USA) using a tissue grinding machine, and total RNA was extracted from tissues according to the manufacturer's instructions. To extract cellular RNA, 1 mL of TRIzol reagent was added to each sample (about 3 × 10^5^–5 × 10^5^ cells) and total RNA was extracted according to the manufacturer's instructions. 1 *μ*g total RNA was reversely transcribed to cDNA. QRT-PCR was performed using iTaq Universal SYBR Green Supermix (BioRad, USA) to measure snoRNA expression level. SnoRNA U6 was used as an internal reference gene and the expression levels of target genes were calculated using the 2^-∆∆Ct^ formula. Three replicates were set up for each sample. Primer sequences are shown in Table S2.

### 2.4. Lentivirus Infection

Lentiviruses were constructed by Shanghai JiKai Gene Medicine Technology Co., Ltd (Table S3). After cells were cultured in plates for 24 hours, lentiviruses were added into the medium using 10 multiplicity of infection (MOI). 24 hours after infection, the medium was refreshed with 1 mL of complete medium. 72 hours after infection, cells were cultured in selective medium containing puromycin (2 *μ*g/mL) for 5 days. Finally, the stably infected cells were continuously cultured in medium with 0.67 *μ*g/mL puromycin.

### 2.5. Cell Proliferation

The Cell Counting Kit-8 (CCK-8) assay (Dojindo, Japan) was used to cell proliferation analysis. 2,000 cells/well were cultured in 96-well plates; then, cell viability was measured by absorbance at 450 nm in 0, 1, 2, 3, 4, and 5 days, 5 replicates in each group.

### 2.6. Colony Formation

1,500 cells were cultured in each well of 6-well plates for 10-12 days, the medium was changed every 4 days. Cells were fixed in methanol for 30 minutes and stained with Gimsa for 30 minutes at room temperature.

### 2.7. Bioinformatics Database

Clinical datasets of CRC were downloaded from The Cancer Genome Atlas (TCGA) (https://portal.gdc.cancer.gov/). SnoRNAs expression profiles in this study were downloaded from SnoRNA in Cancers (SNORic) (http://bioinfo.life.hust.edu.cn/SNORic) [11].

### 2.8. Statistical Analysis

Data were expressed as mean ± standard deviation, three or more parallel replicates were set up for all experiments. The associations between gene expression and CRC clinicopathological parameters were analyzed using *χ*^2^ test. Survival curves were plotted using the Kaplan-Meier method and differences between two survival groups were statistically analyzed by log-rank test. The association between gene expression and prognosis of CRC patient cohorts was assessed using Cox proportional hazards regression model (coxph). Two-tailed Student's *t*-test was used to analyze the data from qRT-PCR assay and colony formation assay. Two-way ANOVA method was used to analyze the data from proliferation assay. Statistical analysis and graphing were performed using SPSS software version 24.0 or GraphPad Prism version 7.0. *p* < 0.05 was considered statistically significant.

## 3. Results

### 3.1. Expressions of SNORD15B, SNORD48, and SNORA5C Were Upregulated in CRC Tissues

**Figure 1:**
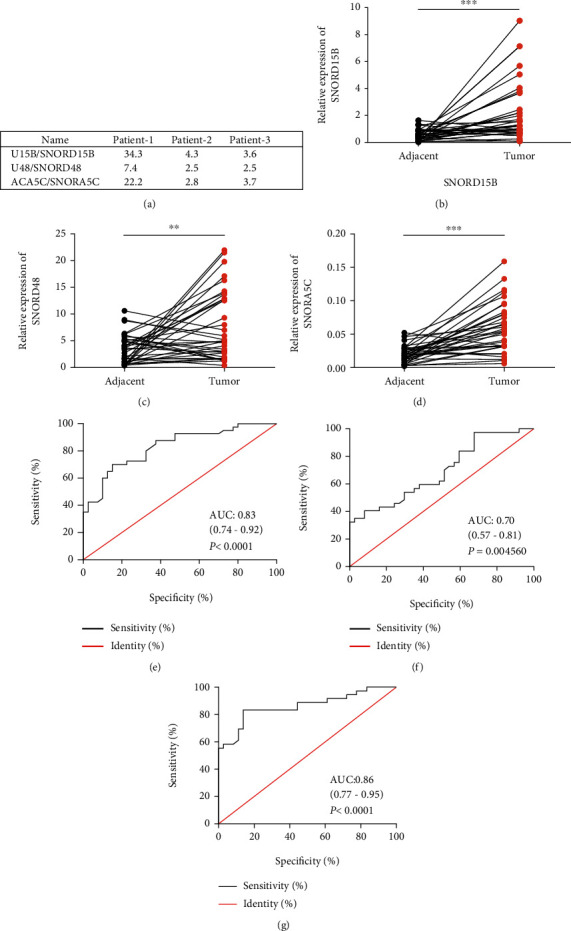
Expressions of SNORD15B, SNORD48, and SNORA5C were upregulated in CRC tissues. (a) Expression levels of SNORD15B, SNORD48, and SNORA5C in 3 paired CRC tissues. (b–d) SNORD15B, SNORD48, and SNORA5C were upregulated in CRC tissues versus adjacent normal mucosa tissues (Paired Student's *t*-test (two-tailed), *n* = 41, ^∗∗^*p* < 0.01, ^∗∗∗^*p* < 0.001). (e–g) ROC curves indicated that SNORD15B, SNORD48, and SNORA5C were differentially expressed in cancer and paraneoplastic tissues.

To reveal snoRNAs' biological effects which might be involved in CRC progression, RNA-seq was employed to screen abnormally expressed snoRNAs for three pairs of CRC and adjacent normal mucosa tissues. We found that some snoRNAs were differentially expressed in cancerous tissues (detailed data not shown). We noticed that the three snoRNA genes SNORD15B, SNORD48, and SNORA5C, biological functions of which had not been reported in CRC, were significantly upregulated in cancerous tissues (Figure 1(a)). Subsequently, to verify whether there were differences in the expression of the genes between cancerous and adjacent normal mucosa tissues from CRC patients, 41 pairs of cancerous/adjacent normal tissues were tested by qRT-PCR. The results showed that expression levels of SNORD15B, SNORD48, and SNORA5C were significantly higher in cancerous tissues versus paracancerous tissues (Figures 1(b)–1(d)). Receiver operating characteristic (ROC) curve analysis indicated that the expression of the three genes significantly discriminated CRC from adjacent normal mucosa tissues (Figures 1(e)–1(g)). Beyond this, it was surprised that these genes were upregulated in kinds of malignant tumors according to the data from SNORic (Figure S1). These results suggested that SNORD15B, SNORD48, and SNORA5C might be potential diagnostic biomarkers for CRC.

### 3.2. High Levels of SNORD15B, SNORD48, and SNORA5C Were Associated with Poor Prognosis in CRC Patients

**Figure 2:**
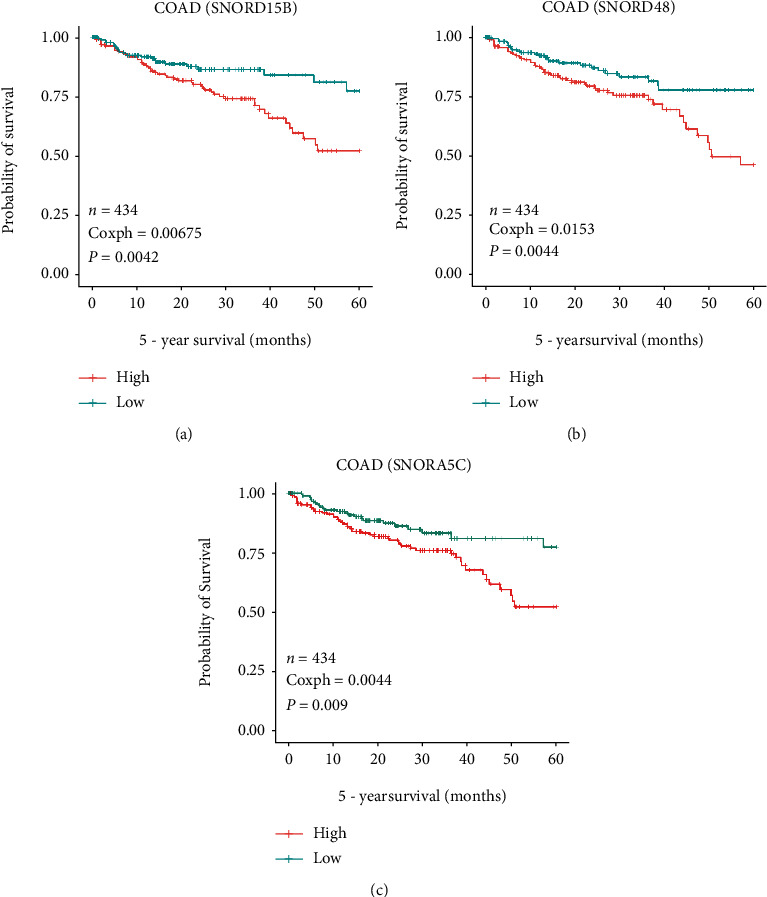
Association between the levels of SNORD15B, SNORD48, and SNORA5C and survival of COAD. To search SNORD15B (a), SNORD48 (b), and SNORA5C (c) in SNORic (http://bioinfo.life.hust.edu.cn/SNORic). Survival curves were plotted using the Kaplan-Meier method. The median expression levels of target genes were used as the cutoff.

Is there any association between expression of these snoRNAs and prognosis of CRC patients? To confirm our hypothesis, we searched data about the above snoRNAs in SNORic and found that SNORD15B, SNORD48, and SNORA5C were all associated with decreased five-year overall survival in colon adenocarcinoma (COAD) patients (Figure 2). Except that, SNORD15B and SNORA5C also displayed negative effect on prognosis of several other cancers including esophageal carcinoma (ESCA), adrenocortical carcinoma (ACC), sarcoma (SARC), kidney renal clear cell carcinoma (KIRC), and thyroid carcinoma (THCA) (Figure S2). The findings suggested that SNORD15B, SNORD48, and SNORA5C might be hidden foes in CRC treatment.

### 3.3. SNORD15B and SNORA5C Promoted Proliferation and Colony Formation in CRC Cells

**Figure 3:**
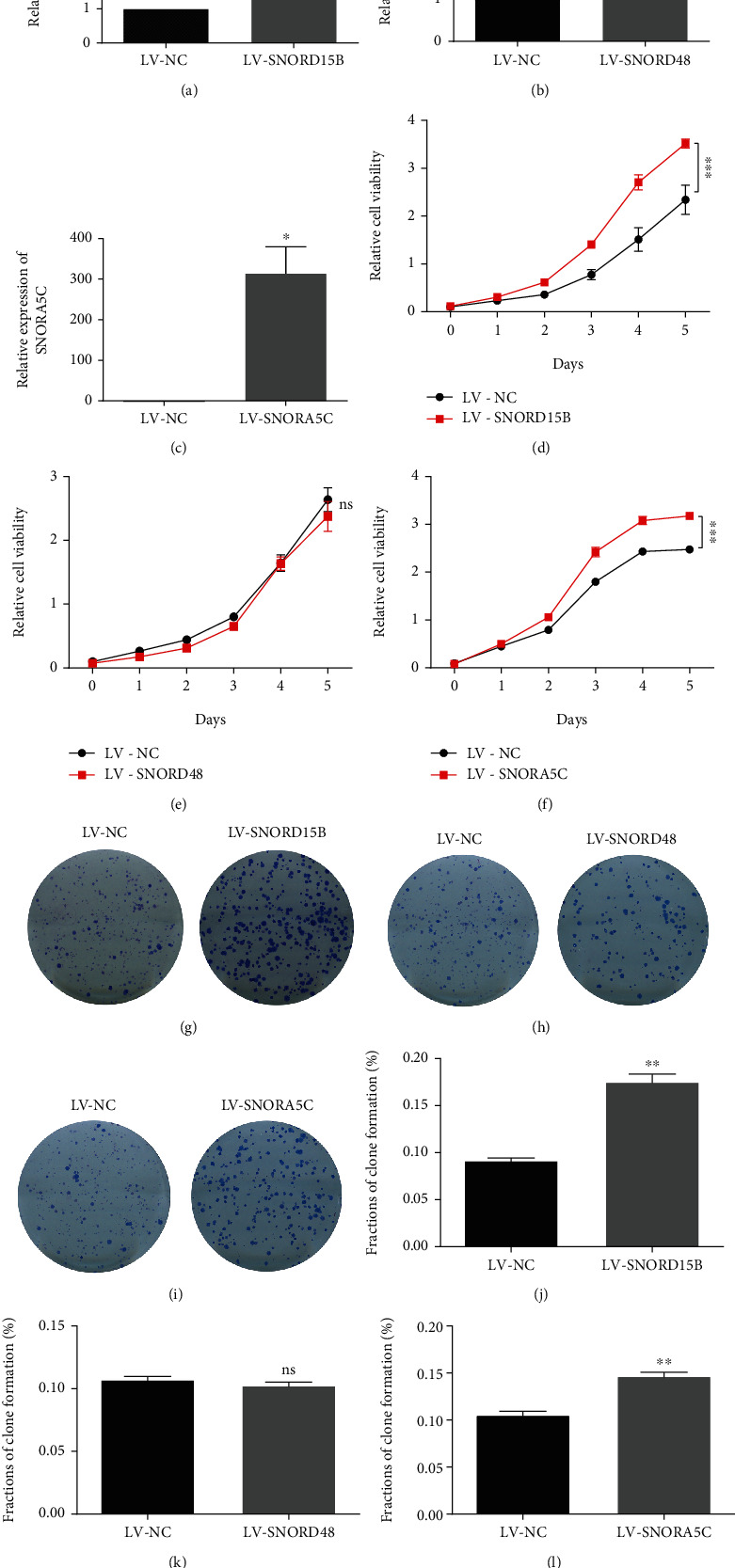
Forced expression of SNORD15B and SNORA5C promoted proliferation and colony formation in HT29 cells. (a–c) The expression of SNORD15B, SNORD48, and SNORA5C in HT29 cells via qRT-PCR test (Two-tailed Student's *t*-test, mean ± SD, *n* = 3). (d–f) Effects of SNORD15B, SNORD48, and SNORA5C on cells proliferation tested by CCK-8 assay (Two-way ANOVA, mean ± SD, *n* = 5). (g–l) Effects of SNORD15B, SNORD48, and SNORA5C on colony formation (Two-tailed Student's *t*-test, mean ± SD, *n* = 3). ^∗^*p* < 0.05, ^∗∗^*p* < 0.01, ^∗∗∗^*p* < 0.001. ns means no significance.

To further validate the roles of SNORD15B, SNORD48, and SNORA5C in CRC, we firstly measured the expression of the genes in human CRC cells (HT29, HCT116, and SW620) and normal colorectal epithelial cells FHC. The data showed that levels of the candidate snoRNAs were not always higher in CRC cells (Figure S3). In the present study, HT29 and HCT116 cells were employed to carry out proliferation and colony formation assay. It was verified that SNORD15B, SNORD48, and SNORA5C were significantly upregulated after lentivirus infection (Figures 3(a)–3(c), Figure S4a-c). SNORD15B and SNORA5C promoted proliferation in both HT29 and HCT116 cells. However, SNORD48 had no influence on cell proliferation (Figures 3(d)–3(f), Figure S4d-f). Meanwhile, SNORD15B and SNORA5C also enhanced colony formation, while SNORD48 had no influence on this phenotype (Figures 3(g)–3(l), Figure S4g-l). These results confirmed that SNORD15B and SNORA5C enhanced survival of in vitro CRC cells.

### 3.4. High Levels of SNORD15B and SNORA5C Were Associated with Tumorigenesis and Metastasis of CRC

To understand the roles of SNORD15B and SNORA5C in CRC progression, correlations between the levels of SNORD15B/SNORA5C and clinicopathological parameters of CRC patients were analyzed based on the data from TCGA (Table S4). The CRC cohort was divided into 2 groups based on the cutoff threshold of gene expression (median value) in all patients. We assessed the association between expression levels of SNORD15B/SNORA5C and pathological characteristics of CRC patients. As shown in Table 1, SNORD15B expression was significantly associated with age, lymphatic invasion, and history of colon polyps. Individuals who were older than 70 years with lymphatic invasion or history of colon polyps had higher level of SNORD15B. Similarly in Table 2, SNORA5C expression was also significantly associated with the abovementioned clinicopathological parameters. It is commonly reported that old age and history of colon polyps are essential risk factors for CRC tumorigenesis. Lymphatic invasion and metastasis can advance the malignant progression of CRC and thus lead to a poor prognosis in patients. Our findings indicated that SNORD15B and SNORA5C might have oncogenic functions in pathological progression of tumorigenesis and metastasis in CRC.

### 3.5. High Levels of SNORD15B and SNORA5C Were Independent Risk Factors for Survival of CRC Patients

To further investigate the potential clinical value of SNORD15B and SNORA5C, univariate and multivariate Cox proportional hazards regression models were applied to analyze several essential factors affecting survival of CRC patients. According to univariate analysis, age, TNM stage, lymph node metastasis, venous infiltration, and high level of SNORD15B were significantly associated with overall survival; while in multivariate analysis, age, TNM stage, and SNORD15B expression level were confirmed as independent risk factors for CRC prognosis (Table 3). For SNORA5C, the result was similar to SNORD15B (Table 4). All these results suggested that high levels of SNORD15B and SNORA5C could predict poorer prognosis of CRC patients. Then would high level of both the genes lead to an even worse outcome? As shown in our data, there was no synergistic effect between the two genes on survival of CRC patients (survival rates in both SNORD15B and SNORA5C high expression group 53% versus others 63%) (Figure S5). All these evidences proved that high expression of SNORD15B or SNORA5C in CRC tissues could independently predict poor prognosis.

## 4. Discussion

SnoRNAs are reported to function as carcinogen in many studies [9, 10, 12–15], but few of them have been well studied in CRC pathological process and majority of these transcripts remain to be defined. In the present study, we demonstrated that SNORD15B, SNORD48, and SNORA5C were upregulated in CRC tissues, significantly discriminating CRC from adjacent normal mucosa tissues. In further investigations, SNORD15B and SNORA5C showed positive effect on proliferation and colony formation in CRC cells. Consistently, via correlation analysis of gene expression and clinicopathological parameters, we found that SNORD15B and SNORA5C were involved in pathological process of CRC and act as independent risk factors for clinical survival.

Dysregulated expressions of snoRNAs in CRC have been reported in several publications since recent years. SnoRNAs SNORA15, SNORA21, SNORA33, SNORA41, SNORA42, SNORA71A, ACA11, etc. are upregulated in CRC, while expression of snoRD123 is downregulated. These genes are expected to be potential biomarkers for cancer diagnosis [10, 16–20]. SNORD15B is a C/D box snoRNA encoded in ribosomal protein S3 gene [19], SNORA5C is a H/ACA box snoRNA encoded in lncRNA SLERT and it is required for SLERT lncRNA biogenesis and nucleolar translocation [16]. Snord15b can be induced by particulate matter 10 (PM10) causing airway inflammation and lung fibrosis in BALB/c mice [21]. And it is upregulated in human CRC tissues in the metastasis versus the benign group according to Koduru et al.'s research [22]. In this study, we found SNORD15B and SNORA5C were upregulated in human CRC tissues versus paired adjacent normal mucosal tissues. Correction: SNORD48 is a C/D box snoRNA used as internal reference for miRNA normalization in cardiac tissue [23]. Contrary to this, the results in our study showed that the expression of SNORD48 was upregulated in CRC.

CRC has long been considered one of the most common and deadly malignancies. The “adenoma-carcinoma sequence” is generally recognized in pathological process of CRC, approximately 15% of colorectal nonmalignant adenomas (also called polyps) are expected to be developed to the carcinoma within about ten years [24]. According to Han et al.'s report, Snord15b responses to PM10 caused lung inflammation in mouse model [21]. Via analyzing the correlation between expression of SNORD15B/SNORA5C and clinicopathological parameters of CRC patient cohorts, we noted that both SNORD15B and SNORA5C were significantly associated with history of colon polyps, which is usually caused by chronic inflammation in colorectum. The finding suggested that SNORD15B and SNORA5C might respond to chronic inflammation occurring in colorectum in the early stage of CRC. To our knowledge, some snoRNAs have effects on proliferation, clonality, migration, and invasion during tumor tumorigenesis [10, 18]. This study showed that SNORD15B and SNORA5C enhanced the proliferation and colony formation in HCT116 and HT29 cells while had no effect on migration and invasion (data not shown). These results together suggested that SNORD15B and SNORA5C may act as oncogenes in CRC progression. We also found that SNORD15B and SNORA5C were significantly associated with lymphatic invasion, a marker of tumor metastasis which is considered one of the most crucial risk factors for CRC survival [25, 26]. Consistent with this, high level of SNORD15B and SNORA5C was proved to be independent prognostic risk factors of overall survival of CRC via both univariate and multivariate Cox regression analysis, which proposed potential prognostic biomarkers for CRC patients. But there was no synergistic effect between the two genes on overall survival of CRC in the present study. It should be noted that expression of SNORD48 also showed significant association with survival of CRC patients, but it showed no effect on cell proliferation and colony formation in vitro experiments.

There were some limitations in our study. Firstly, it was a pity that we had no enough experimental data to identify the biological function of SNORD15B and SNORA5C in other cancers cell lines except CRC, or performed in vivo studies to verify whether they would accelerate tumorigenesis. Secondly, studies about molecular mechanisms of SNORD15B and SNORA5C were still missing. To explore the molecular mechanism of the two genes, transcriptome analysis or proteomic analysis would be employed to cells overexpressing SNORD15B or SNORA5C in future investigations. Undoubtedly, to ensure their potential application prospect in early diagnosis of CRC, more adequate clinical investigations are needed.

In summary，our study demonstrated that SNORD15B, SNORD48, and SNORA5C are upregulated in CRC tissues. Among of them, SNORD15B and SNORA5C have positive effects on proliferation and colony formation in CRC cells. High levels of SNORD15B and SNORA5C are associated with carcinogenesis and metastasis of CRC and they can predict poor prognosis of the patients independently. Our results suggested that SNORD15B and SNORA5C can act as novel potential biomarkers for CRC.

## Figures and Tables

**Table 1:** Correlations between SNORD15B expression and clinicopathological parameters.

Characteristics	Case	SNORD15B expression^#^		*χ* ^2^	*p*
		Low	High		
Sex				0.037	0.847
Female	206	102	104		
Male	224	113	111		
Age (yr)				9.076	0.003^∗^
≤70	230	130	100		
>70	198	83	115		
TNM stage (AJCC)				0.228	0.633
Stage I/II	238	116	122		
Stage III/IV	186	95	91		
Lymphatic invasion				3.901	0.048^∗^
Negative	236	129	107		
Positive	151	67	84		
Venous invasion				0.056	0.813
Negative	278	141	137		
Positive	94	49	45		
History of colon polyps				17.532	< 0.001^∗^
Negative	239	132	107		
Positive	122	39	83		

AJCC = American Joint Committee on Cancer. ^#^Cutoff threshold of SNORD15B expression was median value in all patients in this cohort. ^∗^*p* < 0.05.

**Table 2:** Correlations between SNORA5C expression and clinicopathological parameters.

Characteristics	Case	SNORA5C expression^#^		*χ* ^2^	*p*
		Low	High		
Sex				0.000	1.000
Female	206	103	103		
Male	224	112	112		
Age (yr)				4.174	0.041^∗^
≤70	230	125	105		
>70	198	88	110		
TNM stage (AJCC)				0.486	0.486
Stage I/II	238	122	116		
Stage III/IV	186	89	97		
Lymphatic invasion				12.681	< 0.001^∗^
Negative	236	136	100		
Positive	151	59	92		
Venous invasion				3.656	0.056
Negative	278	150	128		
Positive	94	40	54		
History of colon polyps				9.443	0.002^∗^
Negative	239	127	112		
Positive	122	44	78		

AJCC = American Joint Committee on Cancer. ^#^Cutoff threshold of SNORA5C expression was median value in all patients in this cohort. ^∗^*p* < 0.05.

**Table 3:** Cox regression analysis for SNORD15B and overall survival of CRC.

Variables	HR	Univariate	*p*	HR	Multivariate	*p*
		95% CI			95% CI	
Sex (female vs. male)	1.010	0.669–1.524	0.964	—	—	—
Age (≤ 70 vs. > 70)	1.808	1.188–2.753	0.006^∗^	2.003	1.232–3.255	0.005^∗^
TNM stage (stage I/II vs. stage III/IV)	3.613	2.280–5.726	<0.001^∗^	4.767	2.661–8.540	< 0.001^∗^
Lymphatic invasion (negative vs. positive)	2.256	1.449–3.513	<0.001^∗^	0.848	0.462–1.557	0.595
Venous invasion (negative vs. positive)	2.376	1.505–3.751	< 0.001^∗^	1.696	0.949–3.032	0.075
History of colon polyps (negative vs. positive)	0.768	0.447–1.321	0.340	—	—	—
SNORD15B expression (low vs. high)^#^	1.651	1.081–2.522	0.020^∗^	2.198	1.334–3.622	0.002^∗^

^#^Cutoff threshold of SNORD15B expression was median value in all patients in this cohort. ^∗^*p* < 0.05.

**Table 4:** Cox regression analysis for SNORA5C and overall survival of CRC.

Variables	HR	Univariate	*p*	HR	Multivariate	*p*
		95% CI			95% CI	
Sex (female vs. male)	1.010	0.669–1.524	0.964	—	—	—
Age (≤ 70 vs. > 70)	1.808	1.188–2.753	0.006^∗^	1.995	1.228–3.243	0.005^∗^
TNM stage (stage I/II vs. stage III/IV)	3.613	2.280–5.726	<0.001^∗^	4.379	2.447–7.836	<0.001^∗^
Lymphatic invasion (negative vs. positive)	2.256	1.449–3.513	<0.001^∗^	0.884	0.471–1.661	0.702
Venous invasion (negative vs. positive)	2.376	1.505–3.751	< 0.001^∗^	1.435	0.785–2.626	0.241
History of colon polyps (negative vs. positive)	0.768	0.447–1.321	0.340	—	—	—
SNORA5C expression (low vs. high)^#^	1.608	1.053–2.456	0.028^∗^	2.128	1.278–3.545	0.004^∗^

^#^Cutoff threshold of SNORA5C expression was median value in all patients in this cohort; ^∗^*p* < 0.05.

## Data Availability

All data is available in the main text and supplementary materials. All models, nucleotide sequences, vectors, or plasmids used in the study are available from the corresponding authors upon reasonable request.

## Supplementary Materials

Supplementary Materials See Figures S1-S5 and Table S1-S4 in the supplementary material for comprehensive image analysis.

## References

[1] Aran V., Victorino A. P., Thuler L. C., Ferreira C. G. (2016). Colorectal cancer: epidemiology, disease mechanisms and interventions to reduce onset and mortality. *Clinical Colorectal Cancer*.

[2] Siegel Rebecca L., Kimberly D., Sauer S. A. M. A. G., Fedewa Lynn F., Butterly Joseph C., Anderson Andrea Cercek R. A. (2020). Colorectal cancer statistics, 2020. *CA: a Cancer Journal for Clinicians*.

[3] Cao M., Li H., Sun D., Chen W. (2020). Cancer burden of major cancers in China: a need for sustainable actions. *Cancer Communications*.

[4] Chen H., Li N., Ren J. (2019). Participation and yield of a population-based colorectal cancer screening programme in China. *Gut*.

[5] Dekker E., Tanis P. J., Vleugels J. L. A., Kasi P. M., Wallace M. B. (2019). Colorectal cancer. *Lancet*.

[6] Gu M. J., Huang Q. C., Bao C. Z. (2018). Attributable causes of colorectal cancer in China. *BMC Cancer*.

[7] Bratkovič T., Božič J., Rogelj B. (2020). Functional diversity of small nucleolar RNAs. *Nucleic Acids Research*.

[8] Romano G., Veneziano D., Acunzo M., Croce C. M. (2017). Small non-coding RNA and cancer. *Carcinogenesis*.

[9] Okugawa Y., Toiyama Y., Toden S. (2017). Clinical significance of SNORA42 as an oncogene and a prognostic biomarker in colorectal cancer. *Gut*.

[10] Yoshida K., Toden S., Weng W. (2017). SNORA21 - an oncogenic small nucleolar RNA, with a prognostic biomarker potential in human colorectal cancer. *eBioMedicine*.

[11] Gong J., Li Y., Liu C. J. (2017). A pan-cancer analysis of the expression and clinical relevance of small nucleolar RNAs in human cancer. *Cell Reports*.

[12] Mei Y. P., Liao J. P., Shen J. (2012). Small nucleolar RNA 42 acts as an oncogene in lung tumorigenesis. *Oncogene*.

[13] Cui L., Nakano K., Obchoei S. (2017). Small nucleolar noncoding RNA SNORA23, up-regulated in human pancreatic ductal adenocarcinoma, regulates expression of spectrin repeat-containing nuclear envelope 2 to promote growth and metastasis of xenograft tumors in mice. *Gastroenterology*.

[14] Tian B., Liu J., Zhang N. (2021). Oncogenic SNORD12B activates the AKT-mTOR-4EBP1 signaling in esophageal squamous cell carcinoma via nucleus partitioning of PP-1*α*. *Oncogene*.

[15] Zhang C., Zhao L. M., Wu H. (2018). C/D-box Snord105b promotes tumorigenesis in gastric cancer via ALDOA/C-Myc pathway. *Cellular Physiology and Biochemistry*.

[16] Ferreira H. J., Heyn H., Moutinho C., Esteller M. (2012). CpG island hypermethylation-associated silencing of small nucleolar RNAs in human cancer. *RNA Biology*.

[17] Yang X., Li Y., Li L., Liu J., Wu M., Ye M. (2017). SnoRNAs are involved in the progression of ulcerative colitis and colorectal cancer. *Digestive and Liver Disease*.

[18] Zhang Z., Tao Y., Hua Q., Cai J., Ye X., Li H. (2020). SNORA71A promotes colorectal cancer cell proliferation, migration, and invasion. *BioMed Research International*.

[19] Tycowski K. T., Shu M. D., Steitz J. A. (1993). A small nucleolar RNA is processed from an intron of the human gene encoding ribosomal protein S3. *Genes & Development*.

[20] Xing Y. H., Yao R. W., Zhang Y. (2017). *SLERT* regulates DDX21 rings associated with Pol I transcription. *Cell*.

[21] Han H., Oh E. Y., Lee J. H., Park J. W., Park H. J. (2021). Effects of particulate matter 10 inhalation on lung tissue RNA expression in a murine model. *Tuberculosis and Respiratory Diseases*.

[22] Koduru S. V., Tiwari A. K., Hazard S. W., Mahajan M., Ravnic D. J. (2017). Exploration of small RNA-seq data for small non-coding RNAs in human colorectal cancer. *Journal of Genomics*.

[23] Masè M., Grasso M., Avogaro L. (2017). Selection of reference genes is critical for miRNA expression analysis in human cardiac tissue. A focus on atrial fibrillation. *Scientific Reports*.

[24] Mármol I., Sánchez-de-Diego C., Pradilla Dieste A., Cerrada E., Rodriguez Yoldi M. J. (2017). Colorectal carcinoma: a general overview and future perspectives in colorectal cancer. *International Journal of Molecular Sciences*.

[25] Rahbari N. N., Carr P. R., Jansen L. (2019). Time of metastasis and outcome in colorectal cancer. *Annals of Surgery*.

[26] Veronese N., Nottegar A., Pea A. (2016). Prognostic impact and implications of extracapsular lymph node involvement in colorectal cancer: a systematic review with meta-analysis. *Annals of Oncology*.

