# Supplementary material for: SNORD15B and SNORA5C: Novel Diagnostic and Prognostic Biomarkers for Colorectal Cancer
Source: Biomed Res Int. 2022 May 9;2022:8260800. doi: 10.1155/2022/8260800 (PMC9110153; doi:10.1155/2022/8260800)
Supplement: Supplementary Materials — See Figures S1-S5 and Table S1-S4 in the supplementary material for comprehensive image analysis. [file 8260800.f1.zip › proofread supplementary figure S1-S5.docx]

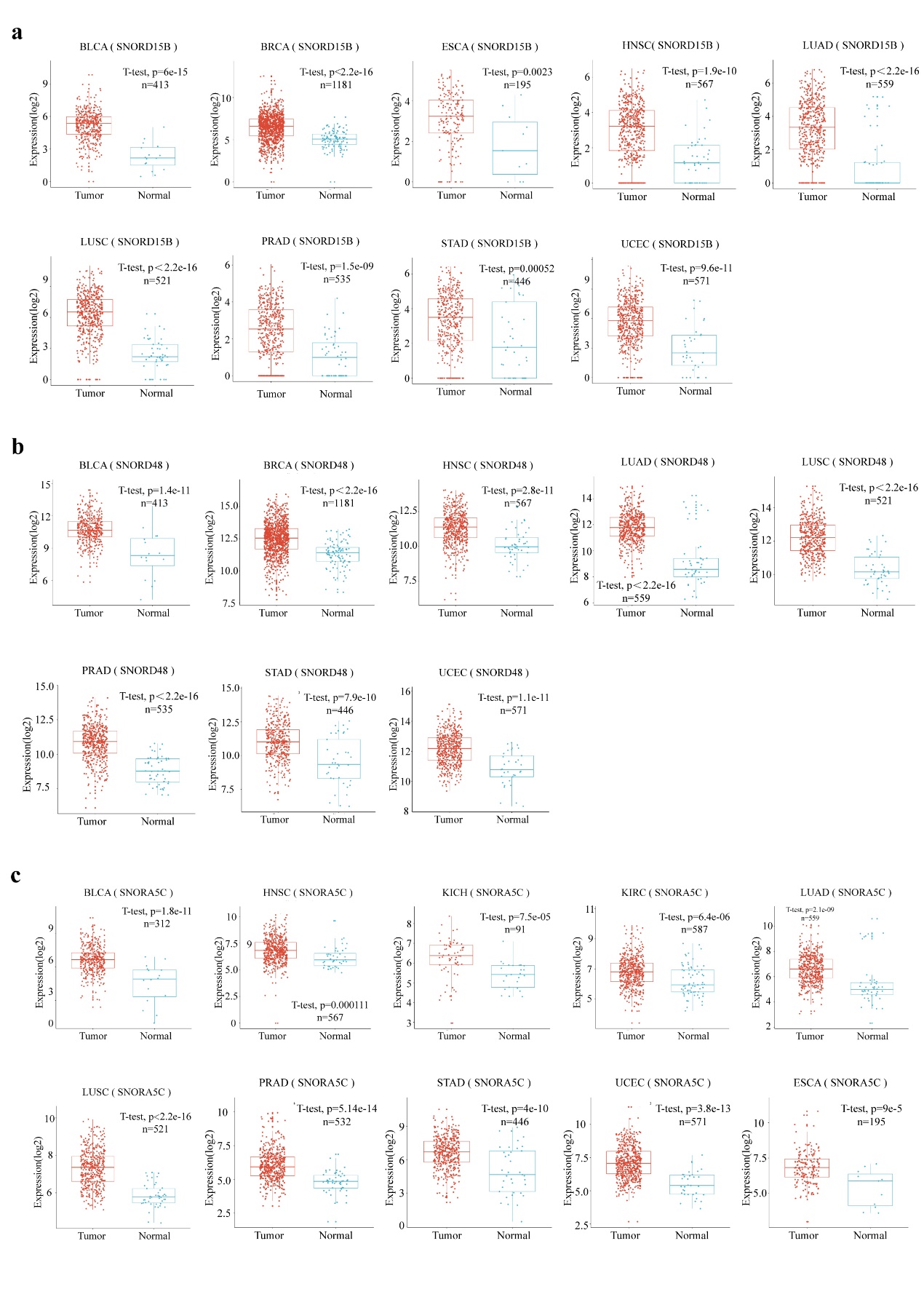


Figure S1: Up-regulated expression of SNORD15B, SNORD48 and SNORA5C in cancers. (a) Expression level of SNORD15B in BLCA (n=41), BRCA (n=1181), ESCA (n=195), HNSC (n=567), LUAD (n=559), LUSC (n=521), PRAD (n=535), STAD (n=446), UCSE (n=571) patients. (b) Expression level of SNORD48 in BLCA (n=41), BRCA (n=1181), HNSC (n=567), LUAD (n=559), LUSC (n=521), PRAD (n=535), STAD (n=446), UCSE (n=571) patients. (c) Expression level of SNORA5C in BLCA (n=41), ESCA (n=195), HNSC (n=567), KICH (n=41), KIRC (n=587), LUAD (n=559), LUSC (n=521), PRAD (n=535), STAD (n=446), UCSE (n=571) patients.


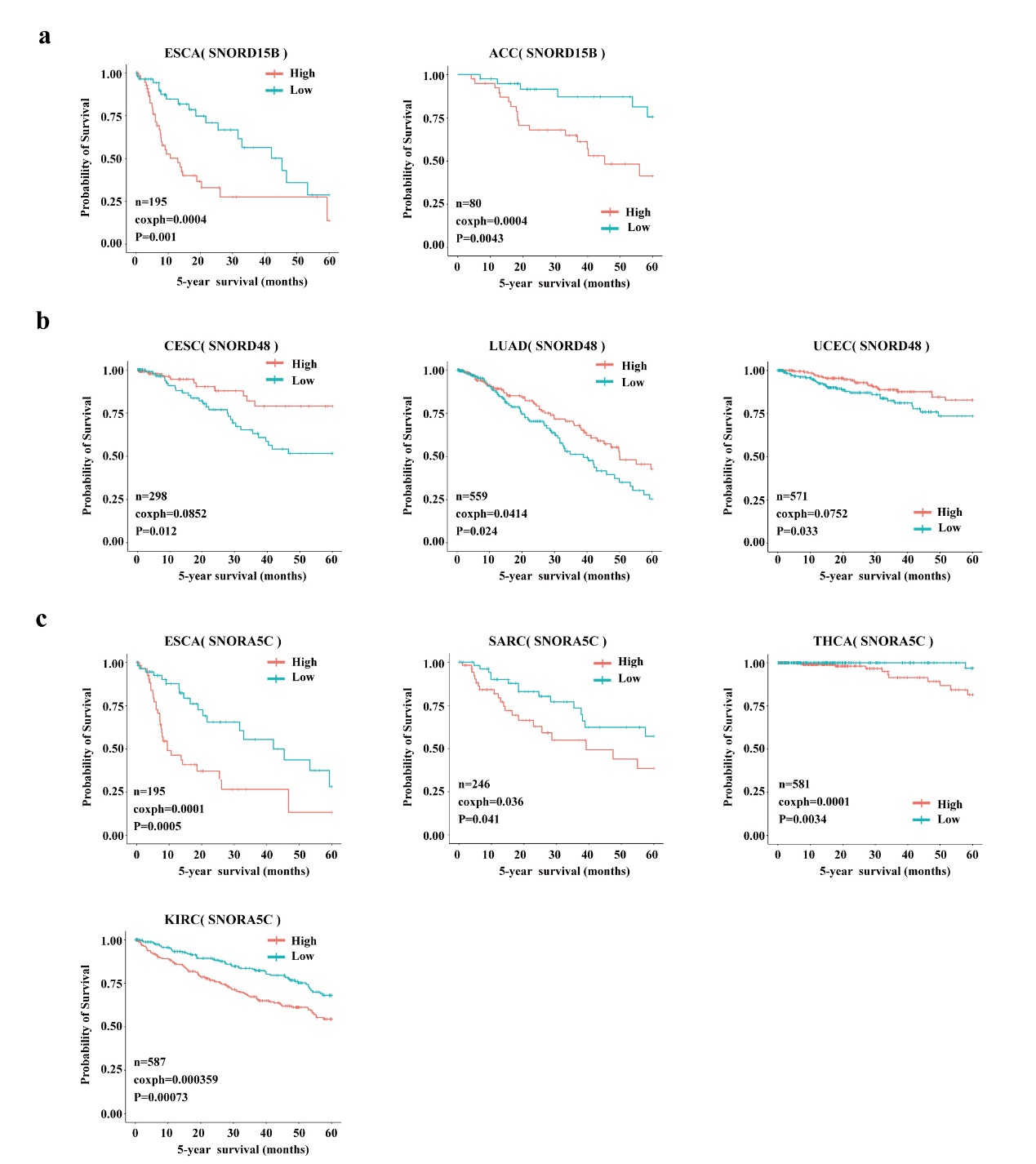


Figure S2: Effects of SNORD15B, SNORD48 and SNORA5C on five-year survival in cancers. Survival curves of overall survival in patients with different cancers was plotted using Kaplan‐Meier method. Cut-off threshold of gene expression was median value: SNORD15B (a), SNORD48 (b) and SNORA5C (c).


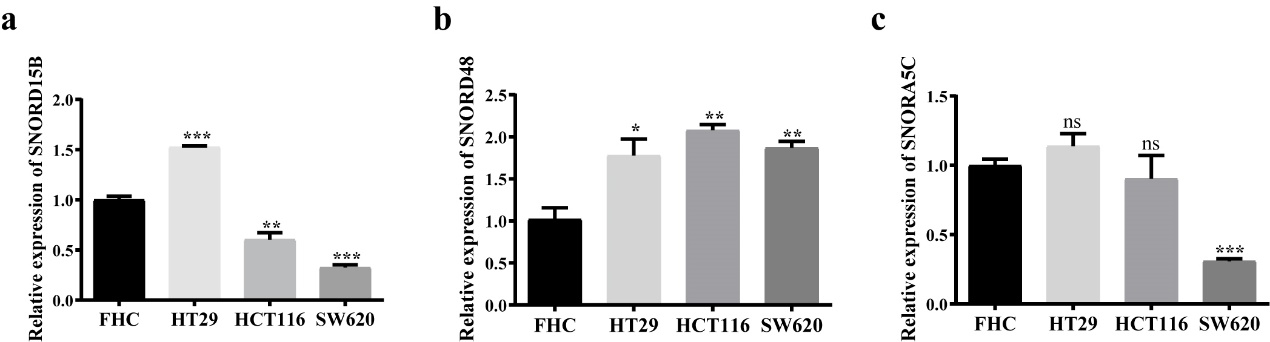


Figure S3: Expression levels of SNORD15B, SNORD48 and SNORA5C in different colorectal epithelial cells. Relative expression levels of SNORD15B (a), SNORD48 (b), SNORA5C (c) in CRC cell lines (HCT116, SW620, and HT29) or normal colon epithelial cell line (FHC) were analyzed by qRT-PCR test (Two-tailed *t*-test, mean ± SD, n = 3). *** P <0.001, ** P <0.01, * P <0.05. ns means no significance.


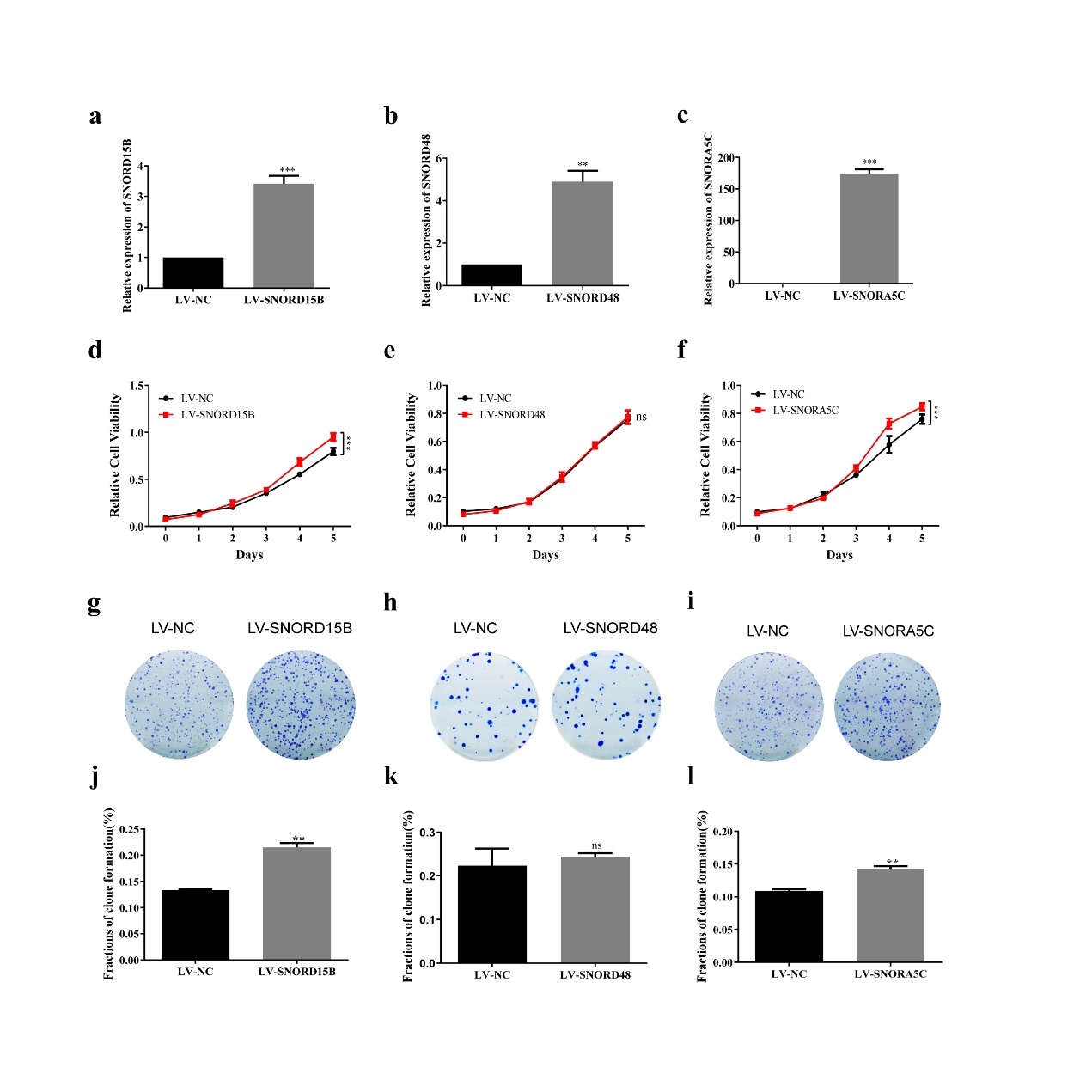
 Figure S4: Forced expression of SNORD15B and SNORA5C promoted proliferation and colony formation in HCT116 cells. (a-c) Expression levels of SNORD15B, SNORD48, SNORA5C in Lentivirus infected HCT116 cell lines. HCT116 cells were infected with 10 MOI LV-SNORD15B, LV- SNORD48, LV- SNORA5C or LV-NC. 72 hours after infection, total RNA was exacted and was performed to qRT-PCR test (Two-tailed Student’s *t*-test, mean ± SD, n = 3). (d-f) Effects of SNORD15B, SNORD48 and SNORA5C on proliferation in HCT116 cells. The infected cells were passaged to 96-well plates in 72 hours after infection, cell viability were measured in 0, 1, 2, 3, 4, 5 days respectively using CCK-8 kit assay, the absorbance values at 450 nm were analyzed (Two-way ANOVA, mean ± SD, n = 5). (g-l) Effects of SNORD15B, SNORD48, SNORA5C on colony formation in HCT116 cells. In colony formation assay, the infected cells were cultured in 6-well plates for 10-12 days, finally fixed in methanol and stained with Gimsa. Triplicates samples in each group. (Two-tailed Student’s *t*-test, mean ± SD, n = 3). *p<0.05, **p<0.01, ***p<0.001, ns means no significance.


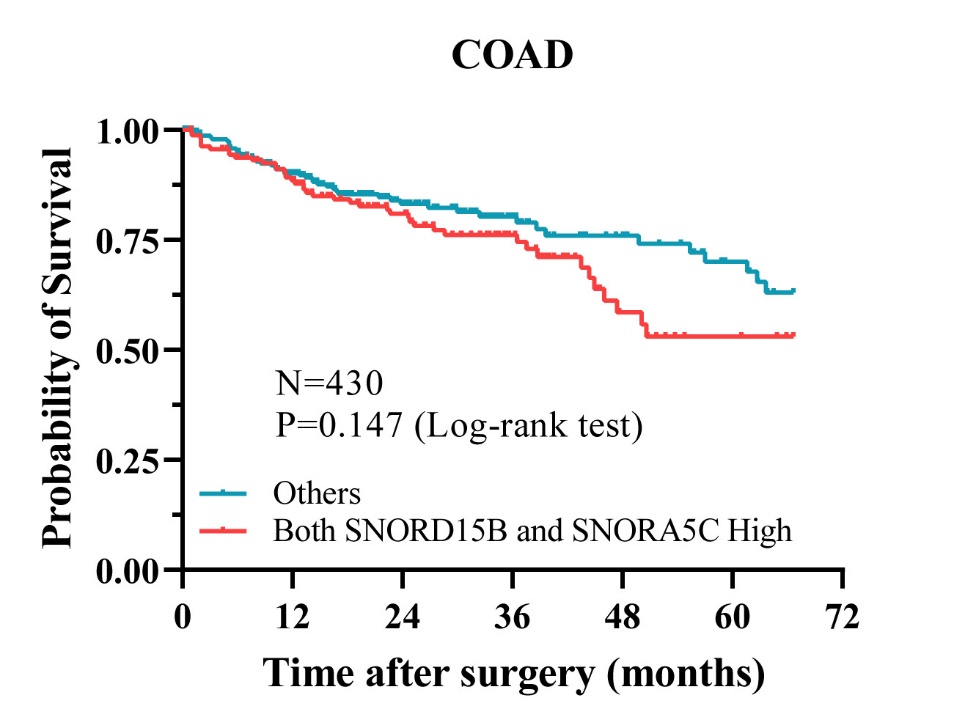


Figure S5: High levels of both SNORD1B and SNORA5C had no [synergistic](javascript:;) [effect](javascript:;) on 5-year overall survival of CRC patients. Survival curves of overall survival in COAD patients was plotted using Kaplan‐Meier method. Patient were divided onto two groups: both high level of SNORD1Band SNORA5C, others (either SNORD1B or SNORA5C high level or both SNORD1B and SNORA5C low level) to estimated cumulative probabilities of overall survival of CRC patients. Cut-off threshold of gene expression was median value in all patients in this cohort.
