# Supplementary material for: SNORD15B and SNORA5C: Novel Diagnostic and Prognostic Biomarkers for Colorectal Cancer
Source: Biomed Res Int. 2022 May 9;2022:8260800. doi: 10.1155/2022/8260800 (PMC9110153; doi:10.1155/2022/8260800)
Supplement: Supplementary Materials — See Figures S1-S5 and Table S1-S4 in the supplementary material for comprehensive image analysis. [file 8260800.f1.zip › Table S1.docx]

| Table S1: Detailed information of patients | | | | | |
| --- | --- | --- | --- | --- | --- |
| Patients ID | Sex | Age(years) | Diagnosis | TNM | Stage |
| 526984 | Female | 76 | rectal cancer | T3N0M0 | IIA |
| 526754 | Female | 60 | rectal cancer | T3N1MO | IIIB |
| 526839 | Male | 46 | rectal cancer | T2N0M0 | I |
| 527300 | Male | 71 | rectal cancer | T2N0M0 | I |
| 527135 | Female | 65 | transverse colon cancer | T4N1M0 | IIIB |
| 527185 | Male | 52 | rectal cancer | T3N0MO | IIA |
| 526214 | Male | 53 | rectal cancer | T2N0M0 | I |
| 526467 | Female | 78 | rectal cancer,hepatic flexure cancer | T3N0M0 | IIA |
| 526820 | Male | 68 | ascending colon cancer | T3N0M0 | IIA |
| 527089 | Female | 58 | rectal cancer | T3N0M0 | IIA |
| 526887 | Male | 71 | rectal cancer | T2N0M0 | I |
| 519396 | Female | 56 | rectal cancer (with liver metastasis) | T3N0M0 | IIA |
| 526900 | Female | 58 | sigmoid colon cancer | T4N1M0 | IIIB |
| 526771 | Male | 81 | ascending colon cancer | T4N2bM0 | IIIC |
| 527549 | Male | 54 | sigmoid colon cancer | T3N0M0 | IIA |
| 526459 | Male | 60 | transverse colon cancer | T3N0M0 | IIA |
| 525862 | Female | 53 | rectal cancer | T2N0M0 | I |
| 525661 | Female | 72 | rectal cancer | T3N1cM0 | IIIB |
| 526146 | Male | 51 | rectal cancer | T2N1aM0 | IIIA |
| 526311 | Male | 67 | ascending colon cancer | T3N0M0 | IIA |
| 526073 | Male | 67 | rectal cancer | T3N0M0 | IIA |
| 525990 | Female | 49 | descending colon cancer | T4aN0M0 | IIB |
| 525831 | Female | 57 | rectal cancer | T2N0MO | I |
| 525744 | Male | 53 | rectal cancer | T3N0MO | IIA |
| 525565 | Female | 41 | sigmoid colon cancer | T3N0M0 | IIA |
| 525615 | Male | 55 | hepatic flexure of colon cancer | T3N0M0 | IIA |
| 525561 | Male | 61 | sigmoid colon cancer | T4aN1bM0 | IIIB |
| 525658 | Male | 63 | rectal cancer | T3N0M0 | IIA |
| 525829 | Female | 60 | hepatic flexure of colon cancer | T4aN1bM0 | IIIB |
| 525789 | Male | 57 | rectal cancer | T3N0M0 | IIA |
| 525304 | Female | 46 | sigmoid colon cancer(with liver metastasis) | yT3N1aM1a | IVA |
| 525813 | Female | 68 | transverse colon cancer | T1N0M0 | I |
| 525298 | Male | 74 | ascending colon cancer | T2N0M0 | I |
| 525402 | Male | 69 | rectal cancer | T4aN2bM0 | IIIC |
| 524672 | Male | 71 | rectal cancer | T3N1bM0 | IIIB |
| 527164 | Female | 77 | rectal cancer | T3N0M0 | IIA |
| 527099 | Male | 43 | rectal cancer | T3N1M0 | IIIB |
| 526332 | Male | 78 | sigmoid colon cancer | T3N0M0 | IIA |
| 527139 | Female | 69 | rectal cancer | T3N0M0 | IIA |
| 527010 | Male | 62 | rectal cancer | T2N0M0 | I |
| 527059 | Female | 54 | sigmoid colon cancer | T3N0M0 | IIA |
