# Supplementary material for: SNORD15B and SNORA5C: Novel Diagnostic and Prognostic Biomarkers for Colorectal Cancer
Source: Biomed Res Int. 2022 May 9;2022:8260800. doi: 10.1155/2022/8260800 (PMC9110153; doi:10.1155/2022/8260800)
Supplement: Supplementary Materials — See Figures S1-S5 and Table S1-S4 in the supplementary material for comprehensive image analysis. [file 8260800.f1.zip › Table S2 (1).docx]

Table S2: Oligonucleotide Sequences of Primers

| Primers | 5’-3’ |
| --- | --- |
| SNORD15B forward | GTCACGTCCTGCTCTTGGTC |
| SNORD15B reverse | CACTTCTGCCAAAGGAACTCG |
| SNORD48 forward | AGTGATGATGACCCCAGGTAACTC |
| SNORD48 reverse | CAGAGCGCTGCGGTGATG |
| SNORA5C forward | TTCAGTGCCCGTTTCTGTCATA |
| SNORA5C reverse | CAAACTTATCCCCAGGTCCCAG |
| U6 forward | CTCGCTTCGGCAGCACA |
| U6 reverse | AACGCTTCACGAATTTGCGT |
