# Supplementary material for: SNORD15B and SNORA5C: Novel Diagnostic and Prognostic Biomarkers for Colorectal Cancer
Source: Biomed Res Int. 2022 May 9;2022:8260800. doi: 10.1155/2022/8260800 (PMC9110153; doi:10.1155/2022/8260800)
Supplement: Supplementary Materials — See Figures S1-S5 and Table S1-S4 in the supplementary material for comprehensive image analysis. [file 8260800.f1.zip › Table S3 (1).docx]

Table S3: Oligonucleotide Sequences of Lentiviruses

| Name | 5’-3’ |
| --- | --- |
| negative control (NC) | TTCTCCGAACGTGTCACGT |
| SNORD15B | CTTCAGTGATGACACGATGACGAGTCAGAAAGGTCACGTCCTGCTCTTGTCCTTGTCAGTGCCATGTTCTGTGGTGCTGTGGCACGAGTTCCTTTGGCAGAAGTGTCCTATTTATTGATCGATTTAGAGGCATTTGTCTGAGAAGG |
| SNORD48 | AGTGATGATGACCCCAGGTAACTCTGAGTGTGTCGCTGATGCCATCACCGCAGCGCTCTGACC |
| SNORA5C | TGCAGTCAAGTCAAATTCAGTGCCCGTTTCTGTCATAGCGGGGGCTGGCCCAGATGGCTGCCACAGCAAGCTCCACAGCTCATGGGCCCTGGGTCACCTACCCTGGGACCTGGGGATAAGTTTGGCTGTGGACAGTG |
