# Supplementary material for: SNORD15B and SNORA5C: Novel Diagnostic and Prognostic Biomarkers for Colorectal Cancer
Source: Biomed Res Int. 2022 May 9;2022:8260800. doi: 10.1155/2022/8260800 (PMC9110153; doi:10.1155/2022/8260800)
Supplement: Supplementary Materials — See Figures S1-S5 and Table S1-S4 in the supplementary material for comprehensive image analysis. [file 8260800.f1.zip › Table S4.docx]

| Table S4: Data from TCGA | | | | | | | | | | |
| --- | --- | --- | --- | --- | --- | --- | --- | --- | --- | --- |
| SampleID | SNORD15B | SNORA5C | Days_to_birth | Sex | Pathologic_stage | Lymphatic_invasion | Venous_invasion | History_of_colon_polyps | _OS | Vital_status |
| TCGA-3L-AA1B-01 | 30.07 | 171.05 | -22379 | FEMALE | Stage I | NO | NO | YES | 475 | Alive |
| TCGA-4N-A93T-01 | 128.47 | 568.17 | -24523 | MALE | Stage IIIB | NO | NO | NO | 146 | Alive |
| TCGA-4T-AA8H-01 | 86.6 | 173.34 | -15494 | FEMALE | Stage IIA | NO | NO | NO | 385 | Alive |
| TCGA-5M-AAT4-01 | 91.58 | 171.96 | -27095 | MALE | Stage IV |  | YES | NO | 49 | Dead |
| TCGA-5M-AAT6-01 | 74.61 | 257.35 | -14852 | FEMALE | Stage IV | YES | YES | NO | 290 | Dead |
| TCGA-5M-AATE-01 | 209.74 | 258.84 | -27870 | MALE | Stage IIA | NO | NO | NO | 1200 | Alive |
| TCGA-A6-2671-01 | 129.06 | 329.94 | -31329 | MALE | Stage IV | YES | YES | YES | 1331 | Dead |
| TCGA-A6-2672-01 | 83.65 | 770.79 | -30237 | FEMALE | Stage IIIB | YES | YES | YES | 1419 | Alive |
| TCGA-A6-2674-01 | 126.57 | 665.94 | -26292 | MALE | Stage IV | NO | YES | NO | 1331 | Alive |
| TCGA-A6-2675-01 | 25.21 | 92.28 | -28813 | MALE | Stage IIA | NO | NO | NO | 1321 | Alive |
| TCGA-A6-2676-01 | 504.17 | 272.06 | -27403 | FEMALE | Stage IIB | NO | NO | NO | 1305 | Dead |
| TCGA-A6-2677-01 | 234.33 | 400.89 | -25143 | FEMALE | Stage IIIC | YES | NO | NO | 740 | Dead |
| TCGA-A6-2678-01 | 215.73 | 292.04 | -16030 | FEMALE | Stage IIIB | YES | NO | NO | 1286 | Alive |
| TCGA-A6-2679-01 | 275.16 | 476.79 | -26820 | FEMALE | Stage IIB | NO | NO | YES | 1366 | Alive |
| TCGA-A6-2680-01 | 99.21 | 269.48 | -26533 | FEMALE | Stage II | NO | NO |  | 1068 | Alive |
| TCGA-A6-2681-01 | 121.73 | 181.99 | -26929 | FEMALE | Stage IIA | NO | NO | NO | 1387 | Alive |
| TCGA-A6-2682-01 | 85.43 | 251.46 | -25683 | MALE | Stage IV | YES | YES | YES | 424 | Dead |
| TCGA-A6-2683-01 | 160.52 | 173.97 | -20871 | FEMALE | Stage IV | NO | NO | YES | 504 | Dead |
| TCGA-A6-2684-01 | 74.92 | 151.44 | -27708 | FEMALE | Stage I | NO | NO | YES | 1127 | Alive |
| TCGA-A6-2685-01 | 74.05 | 123.13 | -17757 | FEMALE | Stage IIA | NO | NO |  | 1133 | Alive |
| TCGA-A6-2686-01 | 245.1 | 250.5 | -29623 | FEMALE | Stage IIA | NO | NO | NO | 1126 | Dead |
| TCGA-A6-3807-01 | 334.46 | 488.53 | -19624 | FEMALE | Stage IIIC | YES | YES | NO | 1054 | Alive |
| TCGA-A6-3808-01 | 208.38 | 191.35 | -26666 | MALE | Stage IIA | NO | NO | YES | 1014 | Alive |
| TCGA-A6-3809-01 | 123.86 | 345.76 | -26274 | FEMALE | Stage IIB | NO | NO | NO | 996 | Alive |
| TCGA-A6-3810-01 | 68.55 | 371.43 | -22999 | MALE | Stage IIA | NO | NO | YES | 1111 | Alive |
| TCGA-A6-4105-01 | 115.88 | 186.44 | -29097 | MALE | Stage IIA | NO | NO | NO | 442 | Dead |
| TCGA-A6-4107-01 | 74.18 | 173.84 | -21064 | FEMALE | Stage IIIB | YES | NO | NO | 987 | Alive |
| TCGA-A6-5656-01 | 154.13 | 736.32 | -27184 | MALE | Stage I | NO | NO | YES | 1001 | Alive |
| TCGA-A6-5657-01 | 18.6 | 33.2 | -23920 | MALE | Stage IIIB | YES | NO | NO | 962 | Alive |
| TCGA-A6-5659-01 | 56.65 | 99.75 | -30028 | MALE | Stage I | NO | NO | NO | 926 | Alive |
| TCGA-A6-5660-01 | 33.19 | 250.54 | -26803 | MALE | Stage IIIC | YES | YES | NO | 888 | Alive |
| TCGA-A6-5661-01 | 38.77 | 56.31 | -29446 | FEMALE | Stage IIA | NO | NO | YES | 1020 | Alive |
| TCGA-A6-5662-01 | 15.81 | 55.12 | -16873 | MALE | Stage IVA | YES | YES | NO | 718 | Alive |
| TCGA-A6-5664-01 | 81.89 | 530.95 | -29426 | MALE | Stage IIIC | YES | YES | YES | 672 | Alive |
| TCGA-A6-5665-01 | 19.19 | 140.19 | -30915 | FEMALE | Stage IIA | YES | NO | NO | 671 | Alive |
| TCGA-A6-5666-01 | 47.71 | 86.97 | -28514 | MALE | Stage IIC | NO | NO | NO | 995 | Alive |
| TCGA-A6-5667-01 | 11.83 | 30.25 | -14756 | FEMALE | Stage IIIB | YES | NO |  | 887 | Alive |
| TCGA-A6-6137-01 | 229.38 | 482.6 | -20095 | MALE | Stage IIIB | NO | NO | YES | 824 | Alive |
| TCGA-A6-6138-01 | 102.22 | 181.48 | -22523 | MALE | Stage I | NO | NO | NO | 685 | Alive |
| TCGA-A6-6140-01 | 214 | 781.02 | -22835 | MALE | Stage IIA | NO | NO | NO | 734 | Alive |
| TCGA-A6-6141-01 | 177.66 | 660.65 | -11391 | MALE | Stage IIA | NO | NO | YES | 130 | Alive |
| TCGA-A6-6142-01 | 69.89 | 216.82 | -20485 | FEMALE | Stage IVA | YES | YES |  | 763 | Alive |
| TCGA-A6-6648-01 | 105.74 | 163.96 | -20641 | MALE | Stage IVA | NO | NO |  | 766 | Alive |
| TCGA-A6-6649-01 | 62.27 | 179.18 | -24237 | MALE | Stage IIIB | YES | YES | YES | 735 | Alive |
| TCGA-A6-6650-01 | 135.61 | 263.29 | -25516 | FEMALE | Stage IIA | NO | NO | NO | 627 | Alive |
| TCGA-A6-6651-01 | 20.99 | 57.38 | -20110 | FEMALE | Stage IIIB | YES | NO | YES | 662 | Alive |
| TCGA-A6-6652-01 | 287.06 | 251.04 | -21638 | MALE | Stage IVA | NO | NO |  | 751 | Alive |
| TCGA-A6-6653-01 | 135.35 | 155.2 | -29996 | MALE | Stage I | NO | NO | YES | 742 | Alive |
| TCGA-A6-6654-01 | 20.03 | 149.01 | -23953 | FEMALE | Stage IIIB | YES | NO | YES | 726 | Alive |
| TCGA-A6-6780-01 | 65.9 | 913.21 | -27250 | MALE | Stage IIA | NO | NO | YES | 612 | Alive |
| TCGA-A6-6781-01 | 65.82 | 104.14 | -15842 | MALE | Stage IIIC | YES | NO |  | 598 | Alive |
| TCGA-A6-6782-01 | 77.85 | 120.62 | -30012 | MALE | Stage IIB | NO |  | NO | 617 | Alive |
| TCGA-A6-A565-01 | 27.05 | 189.47 | -12526 | FEMALE | Stage IIIC | YES | YES | NO | 494 | Dead |
| TCGA-A6-A566-01 | 240.03 | 1768.79 | -20181 | FEMALE | Stage IIIB | YES |  | NO | 758 | Dead |
| TCGA-A6-A567-01 | 45.02 | 73.28 | -20493 | MALE | Stage IV | YES | NO | NO | 1881 | Dead |
| TCGA-A6-A56B-01 | 62.56 | 147.63 | -20903 | MALE | Stage IIIB | YES | NO | NO | 1711 | Dead |
| TCGA-A6-A5ZU-01 | 63.96 | 190.78 | -21554 | MALE | Stage IIIB | YES | NO | NO | 293 | Alive |
| TCGA-AA-3488-01 | 128.93 | 71.53 | -21519 | MALE | Stage IV |  |  | NO | 153 | Dead |
| TCGA-AA-3489-01 | 37.48 | 111.57 | -27606 | MALE | Stage II |  |  | NO | 214 | Dead |
| TCGA-AA-3492-01 | 75.71 | 95.31 | -32871 | FEMALE | Stage II | NO | NO | NO | 92 | Dead |
| TCGA-AA-3494-01 | 138.35 | 175.01 | -20089 | MALE | Stage IV |  |  | NO | 31 | Alive |
| TCGA-AA-3495-01 | 170.75 | 323.35 | -28886 | MALE | Stage I |  |  | YES | 1127 | Alive |
| TCGA-AA-3496-01 | 78.36 | 273.06 | -30438 | FEMALE | Stage II |  |  | NO | 31 | Alive |
| TCGA-AA-3502-01 | 97.66 | 208.05 | -26724 | MALE | Stage I |  |  | YES | 1065 | Alive |
| TCGA-AA-3506-01 | 59.48 | 190.06 | -28275 | MALE | Stage I |  |  |  | 1765 | Alive |
| TCGA-AA-3509-01 | 78.1 | 281.27 | -19786 | FEMALE | Stage II | NO | NO | NO | 1915 | Alive |
| TCGA-AA-3510-01 | 134.71 | 612.08 | -25902 | MALE | Stage II | NO | NO | YES | 1946 | Alive |
| TCGA-AA-3511-01 | 124.75 | 336.87 | -23407 | MALE | Stage II | YES | NO | NO | 212 | Alive |
| TCGA-AA-3514-01 | 1359.99 | 196.31 | -29738 | FEMALE | Stage I | NO | NO | YES | 31 | Alive |
| TCGA-AA-3516-01 | 902.84 | 668.78 | -27303 | FEMALE | Stage III | NO | NO | YES | 396 | Dead |
| TCGA-AA-3517-01 | 252.21 | 565.29 | -22099 | MALE | Stage IIA | NO | NO | YES | 1186 | Alive |
| TCGA-AA-3518-01 | 560.38 | 152.05 | -29769 | FEMALE | Stage IIA | NO | NO | YES | 31 | Alive |
| TCGA-AA-3519-01 | 82.53 | 527.48 | -23131 | MALE | Stage III | NO | NO | YES | 276 | Alive |
| TCGA-AA-3520-01 | 40.88 | 198.39 | -31531 | FEMALE | Stage II | NO | NO | YES | 731 | Alive |
| TCGA-AA-3522-01 | 151.91 | 414.42 | -24653 | MALE | Stage IIA | YES | NO | YES | 1127 | Alive |
| TCGA-AA-3524-01 | 195.29 | 424.37 | -31197 | MALE | Stage II | NO | NO | YES | 1096 | Alive |
| TCGA-AA-3525-01 | 353.55 | 119.98 | -32871 | MALE | Stage IIIB | NO | NO | NO | 245 | Alive |
| TCGA-AA-3526-01 | 894.34 | 270.7 | -21123 | MALE | Stage I | NO | NO | YES | 580 | Alive |
| TCGA-AA-3527-01 | 1229.37 | 273.79 | -32871 | FEMALE | Stage IIA | NO | NO | YES | 0 | Alive |
| TCGA-AA-3529-01 | 81.5 | 253.2 | -28609 | FEMALE | Stage IIIC | YES | NO | NO | 0 | Dead |
| TCGA-AA-3531-01 | 95.82 | 397.54 | -27514 | FEMALE | Stage IIA | NO | NO | NO | 1035 | Alive |
| TCGA-AA-3532-01 | 48.86 | 147.1 | -23133 | MALE | Stage IIA | NO | NO | NO | 882 | Alive |
| TCGA-AA-3534-01 | 80.45 | 186.98 | -28489 | FEMALE | Stage IIA | YES | NO | YES | 882 | Alive |
| TCGA-AA-3538-01 | 194.27 | 305.36 | -19752 | FEMALE | Stage I | NO | NO | NO | 791 | Alive |
| TCGA-AA-3542-01 | 245.54 | 375.98 | -25324 | MALE | Stage IIIC | YES | NO | YES | 395 | Alive |
| TCGA-AA-3543-01 | 420.29 | 180.98 | -30772 | MALE | Stage I | NO | NO | YES | 30 | Alive |
| TCGA-AA-3548-01 | 2211.52 | 189.34 | -26175 | FEMALE | Stage IIIC | YES | NO | NO | 1034 | Alive |
| TCGA-AA-3549-01 | 271.7 | 525.24 | -25415 | MALE | Stage I | NO | NO | YES | 639 | Alive |
| TCGA-AA-3552-01 | 129.46 | 503.65 | -31290 | MALE | Stage IIIC | YES | NO | NO | 396 | Dead |
| TCGA-AA-3553-01 | 76.82 | 299 | -22524 | FEMALE | Stage I | NO | NO | YES | 730 | Alive |
| TCGA-AA-3555-01 | 174.38 | 755.39 | -29617 | FEMALE | Stage IIA | NO | NO | YES | 911 | Alive |
| TCGA-AA-3556-01 | 183.33 | 189.37 | -28704 | MALE | Stage I | NO | NO | YES | 700 | Alive |
| TCGA-AA-3560-01 | 3018.26 | 256.78 | -26359 | FEMALE | Stage IIIC | YES | NO | YES | 608 | Alive |
| TCGA-AA-3561-01 | 138.94 | 372.54 | -26420 | MALE | Stage IIA | NO | NO | YES | 424 | Alive |
| TCGA-AA-3562-01 | 62.69 | 193.66 | -30254 | MALE | Stage IIIC | YES | NO | YES | 608 | Alive |
| TCGA-AA-3655-01 | 24.3 | 101.17 | -24896 | MALE | Stage II | NO | NO | YES | 1856 | Alive |
| TCGA-AA-3660-01 | 29.25 | 191.93 | -18932 | FEMALE | Stage II | NO | NO | NO | 2375 | Alive |
| TCGA-AA-3662-01 | 20.33 | 170.02 | -29554 | FEMALE | Stage IV | NO | NO | YES | 184 | Alive |
| TCGA-AA-3663-01 | 17.61 | 62.53 | -15675 | MALE | Stage II | NO | NO | NO | 212 | Alive |
| TCGA-AA-3664-01 | 135.68 | 762.07 | -27363 | FEMALE | Stage II | NO | NO | NO | 1643 | Alive |
| TCGA-AA-3666-01 | 134.01 | 546.17 | -25020 | MALE | Stage III | NO | NO | NO | 61 | Dead |
| TCGA-AA-3667-01 | 158.93 | 517.29 | -13302 | FEMALE | Stage I | NO | NO | NO | 426 | Alive |
| TCGA-AA-3672-01 | 158.77 | 435.51 | -32873 | FEMALE | Stage III | NO | NO | NO | 28 | Alive |
| TCGA-AA-3673-01 | 491.54 | 589.87 | -19509 | FEMALE | Stage II | NO | NO | NO | 1522 | Alive |
| TCGA-AA-3678-01 | 297.44 | 325.28 | -22219 | FEMALE | Stage III | YES | NO | NO | 1430 | Alive |
| TCGA-AA-3679-01 | 882.51 | 466.15 | -21611 | MALE | Stage IV | YES | NO | NO | 457 | Alive |
| TCGA-AA-3680-01 | 203.04 | 599.27 | -24655 | FEMALE | Stage IV | YES | NO | NO | 335 | Dead |
| TCGA-AA-3681-01 | 165.52 | 574.17 | -28399 | FEMALE | Stage III | NO | NO | YES | 182 | Alive |
| TCGA-AA-3684-01 | 126.48 | 263.84 | -23741 | FEMALE | Stage IV | YES | NO | YES | 0 | Alive |
| TCGA-AA-3685-01 | 213.36 | 566.45 | -25506 | MALE | Stage II | YES | NO | YES | 1127 | Alive |
| TCGA-AA-3688-01 | 247.05 | 670.45 | -29220 | MALE | Stage IV | YES | NO | NO | 578 | Alive |
| TCGA-AA-3692-01 | 237.79 | 442.33 | -17167 | FEMALE | Stage IV | NO | NO | YES | 1095 | Dead |
| TCGA-AA-3693-01 | 268.83 | 865.51 | -28459 | FEMALE | Stage IV | YES | NO | NO | 0 | Alive |
| TCGA-AA-3695-01 | 549.25 | 387.3 | -23164 | FEMALE | Stage IV | YES | NO | YES | 0 | Alive |
| TCGA-AA-3696-01 | 162 | 467.18 | -27667 | FEMALE | Stage IV | NO | NO | YES | 153 | Dead |
| TCGA-AA-3697-01 | 48.63 | 189.95 | -28367 | MALE | Stage II |  |  | NO | 2587 | Alive |
| TCGA-AA-3710-01 | 138.53 | 243.29 | -29465 | FEMALE | Stage IIA | YES |  | YES | 821 | Alive |
| TCGA-AA-3712-01 | 42.82 | 177.48 | -23831 | MALE | Stage III | YES | YES | NO | 0 | Alive |
| TCGA-AA-3713-01 | 34.31 | 107.5 | -24927 | MALE | Stage IV |  |  | YES | 579 | Alive |
| TCGA-AA-3715-01 | 155.13 | 280.93 | -28428 | MALE | Stage II | YES |  | YES | 579 | Dead |
| TCGA-AA-3811-01 | 165 | 345.24 | -30893 | FEMALE | Stage III | YES | YES | NO | 306 | Dead |
| TCGA-AA-3812-01 | 79.64 | 320.04 | -29980 | FEMALE | Stage IIA | YES |  | NO | 1066 | Alive |
| TCGA-AA-3814-01 | 82.96 | 324.03 | -31380 | FEMALE | Stage IIA | YES | YES | NO | 0 | Alive |
| TCGA-AA-3818-01 | 165.46 | 426.7 | -28763 | FEMALE | Stage IIA | NO | NO | NO | 30 | Dead |
| TCGA-AA-3819-01 | 90.08 | 420.23 | -15280 | FEMALE | Stage IIA | YES | YES | NO | 761 | Alive |
| TCGA-AA-3821-01 | 184.97 | 230.82 | -29706 | FEMALE | Stage I |  |  | YES | 31 | Alive |
| TCGA-AA-3831-01 | 420.45 | 220.74 | -24411 | MALE | Stage IIA | YES | YES | NO | 547 | Alive |
| TCGA-AA-3833-01 | 215.8 | 290.64 | -23011 | FEMALE | Stage IIA | YES | YES | YES | 485 | Alive |
| TCGA-AA-3837-01 | 212.89 | 496.28 | -24655 | MALE | Stage IIA | YES |  | YES | 1186 | Alive |
| TCGA-AA-3842-01 | 670.01 | 321.36 | -18842 | MALE | Stage IIIA | YES | YES | YES | 1126 | Alive |
| TCGA-AA-3844-01 | 70.92 | 483.48 | -28521 | FEMALE | Stage IIIC | YES | YES | NO | 454 | Alive |
| TCGA-AA-3845-01 | 72.73 | 133.83 | -31593 | FEMALE | Stage IIA | YES |  | NO | 0 | Dead |
| TCGA-AA-3846-01 | 219.94 | 197.84 | -27057 | FEMALE | Stage IIA | YES |  | YES | 518 | Alive |
| TCGA-AA-3848-01 | 138.31 | 362.65 | -30194 | FEMALE | Stage IIIC | YES | YES | YES | 306 | Dead |
| TCGA-AA-3850-01 | 75.07 | 221.73 | -27090 | MALE | Stage I | YES |  | YES | 0 | Dead |
| TCGA-AA-3851-01 | 193.37 | 224.55 | -27090 | MALE | Stage IIA | YES | YES | YES | 1006 | Alive |
| TCGA-AA-3852-01 | 189.36 | 351.56 | -32203 | MALE | Stage IIA | YES | YES | YES | 0 | Dead |
| TCGA-AA-3854-01 | 184.09 | 132.95 | -26022 | FEMALE | Stage I | YES | YES | YES | 1096 | Alive |
| TCGA-AA-3855-01 | 133.92 | 354.75 | -26541 | MALE | Stage I | YES | YES | YES | 975 | Alive |
| TCGA-AA-3856-01 | 290.55 | 728.46 | -21670 | MALE | Stage IIA | YES | YES | YES | 30 | Alive |
| TCGA-AA-3858-01 | 1094.7 | 293.4 | -24806 | MALE | Stage I | YES | YES | YES | 945 | Alive |
| TCGA-AA-3861-01 | 708.54 | 297.69 | -26602 | MALE | Stage IIA | YES | YES | NO | 914 | Alive |
| TCGA-AA-3862-01 | 162.31 | 494 | -30163 | MALE | Stage IIA | YES |  | NO | 914 | Alive |
| TCGA-AA-3864-01 | 114.64 | 383.47 | -26237 | MALE | Stage II | NO | NO | NO | 1612 | Alive |
| TCGA-AA-3866-01 | 78.71 | 196.7 | -28672 | FEMALE | Stage I | NO | NO | NO | 518 | Alive |
| TCGA-AA-3867-01 | 89.32 | 283.32 | -27028 | MALE | Stage IV | YES | NO | YES | 731 | Alive |
| TCGA-AA-3869-01 | 205.09 | 649.34 | -28062 | MALE | Stage IV | YES | NO | NO | 822 | Dead |
| TCGA-AA-3870-01 | 142.24 | 216.46 | -26086 | FEMALE | Stage IV | YES | NO | YES | 912 | Alive |
| TCGA-AA-3872-01 | 163.11 | 157.53 | -16651 | MALE | Stage IV | YES | YES | NO | 0 | Alive |
| TCGA-AA-3875-01 | 74.76 | 436.63 | -28640 | FEMALE | Stage I | NO | NO | NO | 549 | Alive |
| TCGA-AA-3877-01 | 431.25 | 227.5 | -30377 | FEMALE | Stage I | NO | NO | YES | 943 | Alive |
| TCGA-AA-3930-01 | 328.01 | 466.3 | -24321 | MALE | Stage IV | YES | YES | NO | 61 | Dead |
| TCGA-AA-3939-01 | 124.23 | 546.61 | -30439 | MALE | Stage IIA | NO | NO | YES | 395 | Alive |
| TCGA-AA-3947-01 | 80.72 | 142.49 | -22035 | FEMALE | Stage IIB | NO | NO | YES | 1004 | Alive |
| TCGA-AA-3949-01 | 51.64 | 245.72 | -32081 | FEMALE | Stage IIIB | NO | NO | NO | 791 | Alive |
| TCGA-AA-3950-01 | 263.37 | 352.76 | -29130 | FEMALE | Stage IIA | NO | NO | NO | 730 | Alive |
| TCGA-AA-3952-01 | 81.19 | 298.77 | -24868 | MALE | Stage IIIC | YES | YES | YES | 61 | Dead |
| TCGA-AA-3956-01 | 150.7 | 365.21 | -24045 | MALE | Stage IIA | NO | NO | YES | 1035 | Alive |
| TCGA-AA-3966-01 | 134.5 | 272.62 | -32537 | FEMALE | Stage IIA | YES | YES | YES | 61 | Alive |
| TCGA-AA-3968-01 | 599.44 | 691.17 | -20089 | FEMALE | Stage I | NO | NO | NO | 669 | Alive |
| TCGA-AA-3970-01 | 174.56 | 642.84 | -23741 | MALE | Stage IIA | NO | NO | YES | 1096 | Alive |
| TCGA-AA-3971-01 | 84.49 | 572.17 | -21518 | MALE | Stage III | NO | NO | NO | 489 | Alive |
| TCGA-AA-3972-01 | 109.89 | 338.39 | -26360 | MALE | Stage IV | NO | NO | NO | 1551 | Alive |
| TCGA-AA-3973-01 | 451.41 | 889.49 | -25536 | MALE | Stage IV | YES | YES | NO | 397 | Alive |
| TCGA-AA-3975-01 | 219.43 | 418.06 | -29310 | MALE | Stage I | NO | NO | NO | 1036 | Alive |
| TCGA-AA-3976-01 | 649.96 | 245.8 | -25599 | MALE | Stage IIIA |  | NO | NO | 791 | Alive |
| TCGA-AA-3979-01 | 212.8 | 311.28 | -30742 | MALE | Stage IIA | NO | NO | NO | 730 | Alive |
| TCGA-AA-3980-01 | 251.49 | 422.47 | -32630 | FEMALE | Stage I | YES | YES | YES | 822 | Alive |
| TCGA-AA-3982-01 | 161.42 | 482.55 | -27608 | MALE | Stage IIIB | YES | YES | YES | 822 | Alive |
| TCGA-AA-3984-01 | 218.89 | 466.33 | -22585 | FEMALE | Stage IIA | YES | YES | YES | 0 | Alive |
| TCGA-AA-3986-01 | 169.92 | 259.84 | -26967 | MALE | Stage I | NO | NO | YES | 580 | Alive |
| TCGA-AA-3989-01 | 152.71 | 202.35 | -30712 | MALE | Stage IV | YES | NO | YES | 242 | Dead |
| TCGA-AA-3994-01 | 107.22 | 254.07 | -25323 | MALE | Stage IIIB | NO | NO | YES | 822 | Alive |
| TCGA-AA-A00D-01 | 154.43 | 358.02 | -25689 | MALE | Stage I | NO | NO | YES | 578 | Alive |
| TCGA-AA-A00J-01 | 171.18 | 507.76 | -29371 | MALE | Stage IIIB | YES | NO | YES | 549 | Alive |
| TCGA-AA-A00L-01 | 387.5 | 2499.58 | -24319 | MALE | Stage IIA | NO | NO | YES | 1157 | Alive |
| TCGA-AA-A00O-01 | 157.14 | 243.48 | -30316 | FEMALE | Stage IIIC | YES | NO | NO | 822 | Alive |
| TCGA-AA-A00U-01 | 108.77 | 374.85 | -18263 | MALE | Stage IIIB | NO | NO | YES | 518 | Alive |
| TCGA-AA-A00W-01 | 162.61 | 441.99 | -29311 | MALE | Stage I | NO | NO | YES | 456 | Alive |
| TCGA-AA-A010-01 | 165.86 | 2350.36 | -16922 | FEMALE | Stage IIB | NO | NO | NO | 1064 | Alive |
| TCGA-AA-A01D-01 | 165.53 | 620.19 | -17318 | FEMALE | Stage IIIC | YES | NO | NO | 334 | Dead |
| TCGA-AA-A01F-01 | 121.32 | 1320.31 | -26298 | MALE | Stage IIIB | YES | NO | YES | 974 | Alive |
| TCGA-AA-A01G-01 | 222.56 | 439.13 | -23133 | MALE | Stage IIA | NO | NO | NO | 365 | Alive |
| TCGA-AA-A01P-01 | 60.63 | 307.23 | -29554 | FEMALE | Stage III | YES |  | NO | 1158 | Dead |
| TCGA-AA-A01R-01 | 171.55 | 696.68 | -17289 | MALE | Stage III | YES |  | NO | 1065 | Alive |
| TCGA-AA-A01S-01 | 176.7 | 353.4 | -17257 | FEMALE | Stage III |  |  | NO | 31 | Alive |
| TCGA-AA-A01T-01 | 132.05 | 724.43 | -23192 | FEMALE | Stage III | YES |  | NO | 1005 | Alive |
| TCGA-AA-A01V-01 | 210.74 | 303.99 | -21792 | MALE | Stage I |  |  | NO | 31 | Alive |
| TCGA-AA-A01X-01 | 215.18 | 881.35 | -29281 | FEMALE | Stage III | YES |  | YES | 791 | Alive |
| TCGA-AA-A01Z-01 | 270.08 | 1740.35 | -24990 | MALE | Stage II | YES |  | YES | 1126 | Alive |
| TCGA-AA-A022-01 | 144.67 | 274.51 | -32446 | FEMALE | Stage II | YES | YES | NO | 0 | Alive |
| TCGA-AA-A024-01 | 86.12 | 374.19 | -29708 | MALE | Stage II |  |  | NO | 1188 | Dead |
| TCGA-AA-A029-01 | 127.39 | 1075.69 | -24686 | MALE | Stage II |  |  | YES | 1581 | Alive |
| TCGA-AA-A02E-01 | 102.73 | 894.05 | -30256 | FEMALE | Stage IV | YES | YES | NO | 90 | Dead |
| TCGA-AA-A02F-01 | 78.52 | 509.01 | -25051 | FEMALE | Stage IV | YES | YES | NO | 1216 | Alive |
| TCGA-AA-A02H-01 | 278.29 | 1130.69 | -27362 | FEMALE | Stage IV | YES | YES | NO | 61 | Dead |
| TCGA-AA-A02J-01 | 405.11 | 839.09 | -25749 | FEMALE | Stage IV | YES | YES | NO | 153 | Dead |
| TCGA-AA-A02K-01 | 279.78 | 1132.51 | -18506 | MALE | Stage IV | YES | YES | NO | 426 | Dead |
| TCGA-AA-A02O-01 | 221.09 | 765.41 | -30288 | MALE | Stage II | YES | NO | NO | 28 | Alive |
| TCGA-AA-A02R-01 | 262.69 | 309.28 | -30834 | FEMALE | Stage IIA | YES | YES | NO | 670 | Dead |
| TCGA-AA-A02Y-01 | 164.31 | 981.05 | -26724 | MALE | Stage I | YES | YES | NO | 1216 | Alive |
| TCGA-AA-A03F-01 | 107.84 | 285.72 | -32873 | FEMALE | Stage III |  |  | NO | 549 | Dead |
| TCGA-AA-A03J-01 | 176.67 | 2756.01 | -23986 | FEMALE | Stage I |  |  | YES | 1246 | Alive |
| TCGA-AD-5900-01 | 19.04 | 32.46 | -24776 | MALE | Stage I | NO |  | NO | 370 | Alive |
| TCGA-AD-6548-01 | 74.72 | 324.14 | -29758 | FEMALE | Stage I | NO |  | YES | 650 | Alive |
| TCGA-AD-6888-01 | 80.36 | 546.13 | -26816 | MALE | Stage IIIB | YES | YES | YES | 472 | Dead |
| TCGA-AD-6889-01 | 44.89 | 95.63 |  | MALE | Stage IIA | NO | NO |  | 2532 | Dead |
| TCGA-AD-6890-01 | 85.17 | 273.71 | -23769 | MALE |  | NO | NO |  | 746 | Alive |
| TCGA-AD-6895-01 | 19.52 | 82.01 | -30879 | MALE | Stage IIIB | YES | YES |  | 763 | Alive |
| TCGA-AD-6899-01 | 72.09 | 251.82 | -30693 | MALE | Stage IIIC | YES | YES | NO | 176 | Dead |
| TCGA-AD-6901-01 | 36.71 | 155.66 | -28579 | MALE |  | NO | NO |  | 682 | Dead |
| TCGA-AD-6963-01 | 68.18 | 432.85 | -21294 | MALE |  | NO | NO |  | 834 | Alive |
| TCGA-AD-6964-01 | 32.92 | 193.65 | -21457 | MALE |  | YES | YES | NO | 331 | Dead |
| TCGA-AD-6965-01 | 50.61 | 168.59 | -22853 | MALE | Stage IIIC | YES | YES |  | 805 | Alive |
| TCGA-AD-A5EJ-01 | 32.77 | 99.3 | -27180 | FEMALE | Stage IIA | NO | NO |  | 0 | Alive |
| TCGA-AD-A5EK-01 | 77.71 | 244.33 | -18731 | MALE | Stage I |  | NO | YES | 500 | Alive |
| TCGA-AM-5820-01 | 47.43 | 66.83 | -21902 | FEMALE | Stage IVA | YES | NO | NO | 14 | Alive |
| TCGA-AM-5821-01 | 88.09 | 187.66 | -24903 | FEMALE | Stage IIA | NO | NO | YES | 28 | Alive |
| TCGA-AU-3779-01 | 27.54 | 179.26 | -29460 | FEMALE | Stage IIA | NO | NO | YES | 441 | Alive |
| TCGA-AU-6004-01 | 27.62 | 144.64 | -25259 | FEMALE | Stage I | NO | NO | YES | 824 | Alive |
| TCGA-AY-4070-01 | 745.07 | 400.58 | -18621 | FEMALE | Stage IIIC | YES | YES | NO | 496 | Dead |
| TCGA-AY-4071-01 | 156.82 | 670.69 | -23320 | FEMALE | Stage I | NO | NO | YES | 29 | Dead |
| TCGA-AY-5543-01 | 61.89 | 326.09 | -23870 | FEMALE | Stage IVA | NO | NO | NO | 1004 | Alive |
| TCGA-AY-6196-01 | 11.64 | 121.89 | -17311 | MALE | Stage IIIC | YES | NO | NO | 6 | Alive |
| TCGA-AY-6197-01 | 41.13 | 95.36 | -21957 | MALE | Stage IIA | NO | NO | NO | 652 | Alive |
| TCGA-AY-6386-01 | 35.35 | 190.14 | -24453 | FEMALE | Stage IIIB | NO | NO | NO | 542 | Alive |
| TCGA-AY-A54L-01 | 61.37 | 240.18 | -27074 | FEMALE | Stage I | NO | NO | NO | 525 | Alive |
| TCGA-AY-A69D-01 | 75.88 | 111.62 | -20299 | FEMALE | Stage IIA | NO | NO | NO | 543 | Alive |
| TCGA-AY-A71X-01 | 73.97 | 127.11 | -19904 | FEMALE | Stage I | NO | NO | NO | 588 | Alive |
| TCGA-AY-A8YK-01 | 89.81 | 232.87 | -16140 | MALE | Stage IVA | YES | YES | NO | 573 | Alive |
| TCGA-AZ-4308-01 | 161.69 | 189.12 | -17321 | FEMALE | Stage IIIB | YES | NO |  | 3324 | Alive |
| TCGA-AZ-4313-01 | 71.68 | 392.67 | -18765 | FEMALE | Stage I | YES | YES |  | 2310 | Alive |
| TCGA-AZ-4315-01 | 82.79 | 449.22 | -22340 | MALE | Stage IIA | NO | NO |  | 1776 | Alive |
| TCGA-AZ-4323-01 | 45.89 | 235.53 | -13755 | MALE | Stage IV |  | YES |  | 43 | Dead |
| TCGA-AZ-4614-01 | 279.12 | 408.44 | -26267 | FEMALE | Stage IVA | YES | YES |  | 172 | Dead |
| TCGA-AZ-4615-01 | 185.75 | 455.1 | -30908 | MALE | Stage IIIB | YES | YES |  | 1002 | Alive |
| TCGA-AZ-4616-01 | 53.01 | 110.05 | -30043 | FEMALE | Stage IV | YES | YES |  | 156 | Dead |
| TCGA-AZ-4681-01 | 248.15 | 440.55 | -28906 | FEMALE | Stage IIA |  |  |  | 3247 | Alive |
| TCGA-AZ-4682-01 | 161.71 | 436.65 | -22409 | MALE | Stage IVA | NO | NO |  | 680 | Dead |
| TCGA-AZ-4684-01 | 195.9 | 365.19 | -18252 | MALE | Stage IVA | YES |  |  | 1977 | Alive |
| TCGA-AZ-5403-01 | 22.42 | 82.27 | -15983 | MALE | Stage II | NO | NO |  | 1910 | Dead |
| TCGA-AZ-5407-01 | 18.67 | 141.45 | -18820 | FEMALE | Stage I | NO | NO |  | 2683 | Alive |
| TCGA-AZ-6598-01 | 228.67 | 987.33 | -28182 | FEMALE | Stage II | NO | NO |  | 1503 | Dead |
| TCGA-AZ-6599-01 | 127.76 | 196.77 | -26438 | MALE | Stage I | NO | NO |  | 206 | Dead |
| TCGA-AZ-6600-01 | 97.09 | 236.39 | -23581 | MALE | Stage IV | YES | YES |  | 368 | Dead |
| TCGA-AZ-6601-01 | 53.59 | 159.68 | -25082 | MALE | Stage II | YES | YES |  | 3042 | Dead |
| TCGA-AZ-6603-01 | 92.15 | 225.06 | -28424 | FEMALE |  | NO | NO |  | 899 | Dead |
| TCGA-AZ-6605-01 | 68.72 | 259.87 | -28485 | MALE | Stage IIIB | YES | YES |  | 159 | Dead |
| TCGA-AZ-6606-01 | 136.17 | 308.96 | -29704 | MALE | Stage IV | YES | YES |  | 357 | Dead |
| TCGA-AZ-6607-01 | 31.52 | 104.91 | -25448 | MALE | Stage IV | YES | YES |  | 97 | Dead |
| TCGA-AZ-6608-01 | 251.42 | 1018.01 | -20094 | FEMALE | Stage IIIA | YES | YES |  | 59 | Dead |
| TCGA-CA-5254-01 | 422.85 | 623.47 | -15415 | FEMALE | Stage IIA |  |  |  | 386 | Alive |
| TCGA-CA-5255-01 | 276.38 | 4043.95 | -16743 | MALE | Stage IIA |  |  |  | 376 | Alive |
| TCGA-CA-5256-01 | 137.92 | 306.94 | -19785 | FEMALE | Stage IIA |  |  | NO | 379 | Alive |
| TCGA-CA-5796-01 | 49.31 | 149.78 | -19353 | FEMALE | Stage IIA |  |  |  | 377 | Alive |
| TCGA-CA-5797-01 | 33.8 | 227.38 | -20662 | MALE | Stage IIA |  |  |  | 383 | Alive |
| TCGA-CA-6715-01 | 59.43 | 206.1 | -23075 | MALE | Stage IIIB |  |  |  | 383 | Alive |
| TCGA-CA-6716-01 | 30.41 | 135.73 | -23943 | MALE | Stage IIA |  |  |  | 371 | Alive |
| TCGA-CA-6717-01 | 18.66 | 58.11 | -20984 | MALE | Stage IIA |  |  |  | 388 | Alive |
| TCGA-CA-6718-01 | 43.07 | 72.71 | -17109 | MALE | Stage IIA |  |  |  | 306 | Dead |
| TCGA-CA-6719-01 | 40.3 | 86.44 | -28343 | MALE | Stage IIA |  |  |  | 435 | Alive |
| TCGA-CK-4947-01 | 18.07 | 68.33 | -16980 | FEMALE | Stage IIIB |  |  | NO | 534 | Alive |
| TCGA-CK-4948-01 | 32.65 | 109.89 | -16736 | FEMALE | Stage III |  |  | NO | 4502 | Alive |
| TCGA-CK-4950-01 | 29.49 | 125.65 | -24944 | FEMALE | Stage IIIB | NO | NO | NO | 2599 | Alive |
| TCGA-CK-4951-01 | 165.46 | 129.25 | -28982 | FEMALE | Stage IIA | NO | YES | YES | 2134 | Dead |
| TCGA-CK-4952-01 | 10.31 | 96.11 | -17826 | FEMALE | Stage IIIC | NO | NO | NO | 475 | Alive |
| TCGA-CK-5912-01 | 81.99 | 258.89 | -29938 | MALE | Stage I | NO | NO | NO | 1493 | Dead |
| TCGA-CK-5913-01 | 20.67 | 55.78 | -21399 | FEMALE | Stage IIA | NO | NO | NO | 1561 | Alive |
| TCGA-CK-5914-01 | 43.23 | 86.68 | -29586 | MALE | Stage IIIB | NO | NO | NO | 304 | Alive |
| TCGA-CK-5915-01 | 67.44 | 138.43 | -23040 | MALE | Stage I | NO | NO | YES | 0 | Alive |
| TCGA-CK-5916-01 | 16.28 | 86.06 | -26024 | FEMALE | Stage I | YES | NO | YES | 643 | Dead |
| TCGA-CK-6746-01 | 109.25 | 102.9 | -30957 | FEMALE | Stage IIB | NO | NO | NO | 0 | Alive |
| TCGA-CK-6747-01 | 116.56 | 68.88 | -32052 | FEMALE | Stage IIA | NO | NO | NO | 2523 | Alive |
| TCGA-CK-6748-01 | 109.58 | 192.63 | -16529 | FEMALE | Stage IV | NO | NO | NO | 58 | Alive |
| TCGA-CK-6751-01 | 80.76 | 105.14 | -32354 | FEMALE | Stage I | NO | NO | NO | 3780 | Alive |
| TCGA-CM-4743-01 | 17.06 | 63.61 | -25291 | MALE | Stage IIA | YES | NO | NO | 701 | Alive |
| TCGA-CM-4744-01 | 187.29 | 431.72 | -25506 | MALE | Stage I | YES | YES | YES | 609 | Alive |
| TCGA-CM-4746-01 | 224.91 | 357.89 | -22403 | MALE | Stage I | NO | NO | YES | 1126 | Alive |
| TCGA-CM-4747-01 | 155.96 | 364.14 | -17198 | MALE | Stage IVA | YES | NO | YES | 761 | Alive |
| TCGA-CM-4748-01 | 136.63 | 330.08 | -19509 | MALE | Stage IIIB | YES | YES | YES | 792 | Alive |
| TCGA-CM-4750-01 | 150.46 | 440.74 | -12478 | FEMALE | Stage IIIA | YES | NO | YES | 244 | Alive |
| TCGA-CM-4751-01 | 34.69 | 146.2 | -22891 | MALE | Stage IIIB | YES | YES | NO | 822 | Alive |
| TCGA-CM-4752-01 | 138.24 | 520.53 | -21519 | MALE | Stage IIA | NO | NO | YES | 396 | Alive |
| TCGA-CM-5341-01 | 146.8 | 273.2 | -30163 | FEMALE | Stage IIIA | YES | NO | YES | 884 | Alive |
| TCGA-CM-5344-01 | 19.54 | 95.49 | -14426 | FEMALE | Stage IIIB | YES | NO | YES | 670 | Alive |
| TCGA-CM-5348-01 | 5.34 | 94.84 | -26543 | MALE | Stage IIIB | YES | NO | YES | 699 | Alive |
| TCGA-CM-5349-01 | 43.73 | 111.17 | -24867 | FEMALE | Stage IIA | NO | NO | NO | 915 | Alive |
| TCGA-CM-5860-01 | 15.72 | 128 | -16344 | MALE | Stage IIA | NO | YES | NO | 974 | Alive |
| TCGA-CM-5861-01 | 13.7 | 52.54 | -23253 | FEMALE | Stage IIA | NO | NO | NO | 457 | Alive |
| TCGA-CM-5862-01 | 42.86 | 203.54 | -29493 | MALE | Stage IVA | NO | YES | NO | 153 | Dead |
| TCGA-CM-5863-01 | 48.36 | 93 | -22127 | FEMALE | Stage IIIB | NO | YES | NO | 457 | Alive |
| TCGA-CM-5864-01 | 79.48 | 383.16 | -22219 | MALE | Stage I | NO | NO | NO | 457 | Alive |
| TCGA-CM-5868-01 | 34.62 | 145.84 | -21672 | FEMALE | Stage IVA | YES | YES | NO | 518 | Alive |
| TCGA-CM-6161-01 | 23.33 | 90.46 | -13363 | FEMALE | Stage I | NO | NO | NO | 457 | Alive |
| TCGA-CM-6162-01 | 14.19 | 42.32 | -17624 | FEMALE | Stage IIIB | YES | YES | NO | 365 | Alive |
| TCGA-CM-6163-01 | 25.29 | 197.88 | -27150 | MALE | Stage I | NO | YES | NO | 427 | Alive |
| TCGA-CM-6164-01 | 22.04 | 96.25 | -16802 | FEMALE | Stage IIA | NO | YES | NO | 883 | Alive |
| TCGA-CM-6165-01 | 21.83 | 90.91 | -27302 | MALE | Stage IIA | NO | NO | NO | 488 | Alive |
| TCGA-CM-6166-01 | 68.48 | 71.96 | -17807 | FEMALE | Stage I | NO | NO | NO | 669 | Alive |
| TCGA-CM-6167-01 | 8.68 | 26.7 | -21031 | FEMALE | Stage IIIC | YES | YES | NO | 456 | Alive |
| TCGA-CM-6168-01 | 7.95 | 29.32 | -30834 | FEMALE | Stage IIA | NO | YES | NO | 395 | Alive |
| TCGA-CM-6169-01 | 64.4 | 228.38 | -24564 | MALE | Stage IIA | YES | YES | NO | 396 | Alive |
| TCGA-CM-6170-01 | 36 | 180.23 | -26663 | FEMALE | Stage I | NO | NO | YES | 457 | Alive |
| TCGA-CM-6171-01 | 21.66 | 73.35 | -28307 | FEMALE | Stage I | NO | NO | NO | 427 | Alive |
| TCGA-CM-6172-01 | 22.72 | 176.34 | -25902 | FEMALE | Stage IIIB | NO | NO | NO | 335 | Alive |
| TCGA-CM-6674-01 | 27.36 | 83.41 | -14368 | MALE | Stage IIA | NO | NO | NO | 394 | Alive |
| TCGA-CM-6675-01 | 79.2 | 183.92 | -12935 | MALE | Stage IVB | NO | YES | NO | 397 | Alive |
| TCGA-CM-6676-01 | 116.11 | 237.63 | -30223 | MALE | Stage I | YES | NO | NO | 337 | Alive |
| TCGA-CM-6677-01 | 137.75 | 415.21 | -27575 | FEMALE | Stage IIA | NO | NO | NO | 337 | Alive |
| TCGA-CM-6678-01 | 93.18 | 606.9 | -23070 | FEMALE | Stage IVA | NO | NO | NO | 335 | Alive |
| TCGA-CM-6679-01 | 33.62 | 201.15 | -21519 | MALE | Stage IIA | NO | NO | NO | 306 | Alive |
| TCGA-CM-6680-01 | 52.62 | 134.95 | -28701 | FEMALE | Stage IIIB | NO | NO | NO | 366 | Alive |
| TCGA-D5-5537-01 | 105.16 | 290.55 | -30505 | MALE | Stage IIA | NO | NO | NO | 1381 | Dead |
| TCGA-D5-5538-01 | 35 | 114.8 | -21974 | FEMALE | Stage IIIB | NO | NO | NO | 1661 | Dead |
| TCGA-D5-5539-01 | 48.28 | 85.72 | -22097 | MALE | Stage IIIA | YES |  |  | 596 | Alive |
| TCGA-D5-5540-01 | 56.27 | 102.25 | -26972 | MALE | Stage IIA | NO | NO | NO | 1706 | Alive |
| TCGA-D5-5541-01 | 53.06 | 145.83 | -23109 | MALE | Stage IIIB | NO | NO | NO | 1701 | Alive |
| TCGA-D5-6529-01 | 86.05 | 189.58 | -25394 | MALE | Stage IIA | NO | NO |  | 614 | Alive |
| TCGA-D5-6530-01 | 20.32 | 134.96 | -19591 | MALE | Stage I | NO | NO |  | 621 | Alive |
| TCGA-D5-6531-01 | 39.73 | 119.59 | -27633 | MALE | Stage IIA | NO | NO | NO | 540 | Alive |
| TCGA-D5-6532-01 | 59.6 | 183.98 | -22344 | MALE | Stage IIA | NO | NO | NO | 555 | Alive |
| TCGA-D5-6533-01 | 53.56 | 172.16 | -24971 | FEMALE | Stage IIC | NO | NO | NO | 775 | Alive |
| TCGA-D5-6534-01 | 32.09 | 109.4 | -22778 | FEMALE | Stage IIA | NO | NO | YES | 1316 | Alive |
| TCGA-D5-6535-01 | 21.13 | 102.72 | -29278 | FEMALE | Stage IIIB | YES | NO | NO | 460 | Alive |
| TCGA-D5-6536-01 | 11.38 | 70.28 | -26890 | MALE | Stage IIA | NO | NO |  | 543 | Alive |
| TCGA-D5-6537-01 | 20.38 | 176.55 | -23381 | MALE | Stage IIIB | YES | NO |  | 146 | Dead |
| TCGA-D5-6538-01 | 31.34 | 127.36 | -29194 | FEMALE | Stage IIIB | NO | YES | NO | 521 | Alive |
| TCGA-D5-6539-01 | 20.3 | 68.2 | -16703 | FEMALE | Stage IIA | NO | NO |  | 380 | Alive |
| TCGA-D5-6540-01 | 29.57 | 89.01 | -24282 | MALE | Stage I | NO | NO | NO | 491 | Alive |
| TCGA-D5-6541-01 | 48.71 | 116.6 | -18029 | MALE | Stage IIA | NO | NO |  | 474 | Alive |
| TCGA-D5-6898-01 | 99.94 | 55.26 | -18874 | FEMALE | Stage I | NO | NO | NO | 229 | Alive |
| TCGA-D5-6920-01 | 48.06 | 102.38 | -28124 | FEMALE | Stage IIA | NO | NO | NO | 377 | Alive |
| TCGA-D5-6922-01 | 43.64 | 41.92 | -27771 | MALE | Stage IIIA | YES | NO | NO | 308 | Alive |
| TCGA-D5-6923-01 | 46.04 | 181.75 | -21118 | MALE | Stage I | NO | NO | NO | 378 | Alive |
| TCGA-D5-6924-01 | 57.09 | 168.41 | -24964 | MALE | Stage IIA | NO | NO | NO | 435 | Alive |
| TCGA-D5-6926-01 | 65.08 | 79.22 | -23768 | MALE | Stage IIIB | NO | NO | NO | 275 | Alive |
| TCGA-D5-6927-01 | 68.06 | 92.85 | -12737 | MALE | Stage IIA | NO | NO | NO | 287 | Alive |
| TCGA-D5-6928-01 | 36.15 | 44.43 | -29411 | MALE | Stage IIA | NO | NO | NO | 354 | Alive |
| TCGA-D5-6929-01 | 253.64 | 317.69 | -17919 | FEMALE | Stage IV | YES | NO | NO | 408 | Alive |
| TCGA-D5-6930-01 | 9.42 | 42.14 | -24764 | MALE | Stage IIA | NO | NO | NO | 406 | Alive |
| TCGA-D5-6931-01 | 73.57 | 91.43 | -28292 | MALE | Stage IIIC | YES | NO | NO | 365 | Alive |
| TCGA-D5-6932-01 | 88.9 | 208.57 | -25466 | MALE | Stage IIA | NO | NO | NO | 346 | Alive |
| TCGA-D5-7000-01 | 38.87 | 63.89 | -28913 | FEMALE | Stage I | NO | NO | NO | 312 | Alive |
| TCGA-DM-A0X9-01 | 233.05 | 187.83 | -26217 | FEMALE | Stage IIA | NO | NO | NO | 3641 | Alive |
| TCGA-DM-A0XD-01 | 232.96 | 904.39 | -23966 | MALE | Stage IIA | NO | NO | NO | 743 | Dead |
| TCGA-DM-A0XF-01 | 140.08 | 785.17 | -25032 | FEMALE | Stage IIIC | NO | NO | NO | 1162 | Dead |
| TCGA-DM-A1D0-01 | 97.44 | 514.84 | -28875 | FEMALE | Stage IIA | NO | NO | NO | 3974 | Alive |
| TCGA-DM-A1D4-01 | 263.2 | 1999.8 | -29403 | MALE | Stage IIA | NO | NO |  | 2821 | Dead |
| TCGA-DM-A1D6-01 | 121 | 380.87 | -32302 | MALE | Stage IIA | NO | NO | NO | 1518 | Dead |
| TCGA-DM-A1D7-01 | 113.45 | 424.14 | -30142 | MALE | Stage IIA | NO | NO |  | 405 | Dead |
| TCGA-DM-A1D8-01 | 280.78 | 180.25 | -18311 | FEMALE |  |  |  | NO | 383 | Dead |
| TCGA-DM-A1D9-01 | 150.96 | 317.7 | -24514 | FEMALE | Stage IIA | NO | NO | NO | 4270 | Alive |
| TCGA-DM-A1DA-01 | 181.38 | 460.73 | -26169 | FEMALE | Stage IIIC |  |  |  | 228 | Dead |
| TCGA-DM-A1DB-01 | 421.39 | 489.67 | -24888 | MALE | Stage IIA | NO | NO |  | 1348 | Dead |
| TCGA-DM-A1HA-01 | 208.18 | 237.79 | -30272 | MALE | Stage IIIC |  |  | YES | 4000 | Alive |
| TCGA-DM-A1HB-01 | 37.94 | 127.55 | -27708 | MALE | Stage IIIB | NO | NO | NO | 4126 | Alive |
| TCGA-DM-A280-01 | 49.02 | 94.95 | -25649 | FEMALE | Stage IIA | NO | NO | NO | 236 | Dead |
| TCGA-DM-A282-01 | 76.48 | 96.82 | -22265 | FEMALE | Stage IIA | NO | NO | NO | 4233 | Alive |
| TCGA-DM-A285-01 | 42.88 | 116.51 | -26021 | FEMALE | Stage IV |  |  | YES | 179 | Dead |
| TCGA-DM-A288-01 | 70.1 | 644.78 | -25084 | MALE | Stage IIIC | NO | NO | NO | 427 | Dead |
| TCGA-DM-A28A-01 | 140.76 | 87.32 | -28833 | MALE | Stage IIIC | NO | NO | NO | 805 | Dead |
| TCGA-DM-A28C-01 | 244.55 | 276.6 | -27073 | MALE | Stage IIA |  |  | NO | 2475 | Dead |
| TCGA-DM-A28E-01 | 188.13 | 547.47 | -26602 | FEMALE | Stage IIA | NO | NO | NO | 3648 | Alive |
| TCGA-DM-A28F-01 | 99.56 | 99.3 | -26856 | MALE | Stage IIIB |  |  | YES | 1094 | Dead |
| TCGA-DM-A28G-01 | 34.58 | 80.49 | -27641 | MALE | Stage IIA | NO | NO | NO | 1849 | Dead |
| TCGA-DM-A28H-01 | 78.06 | 346.06 | -18325 | MALE | Stage IIIC | NO | NO | NO | 3561 | Alive |
| TCGA-DM-A28K-01 | 85.83 | 151.82 | -27428 | MALE | Stage IIA | NO | NO | NO | 2988 | Alive |
| TCGA-DM-A28M-01 | 132.49 | 273.84 | -23043 | MALE | Stage IIA | NO | NO | NO | 2895 | Alive |
| TCGA-F4-6459-01 | 91.79 | 242.14 | -22574 | FEMALE | Stage IIIB | NO | NO | NO | 262 | Dead |
| TCGA-F4-6460-01 | 93.01 | 198.15 | -18637 | FEMALE | Stage IIIB | NO | NO | NO | 972 | Dead |
| TCGA-F4-6461-01 | 93.92 | 250.94 | -15151 | FEMALE | Stage IIIC | NO | NO | NO | 338 | Dead |
| TCGA-F4-6463-01 | 26.65 | 34.07 | -18804 | MALE | Stage IIA | NO | NO |  | 1087 | Alive |
| TCGA-F4-6569-01 | 32.32 | 118.09 | -22203 | MALE | Stage I | NO | NO |  | 1087 | Alive |
| TCGA-F4-6570-01 | 74.29 | 108.24 | -28772 | FEMALE | Stage IIA | NO | NO |  | 188 | Dead |
| TCGA-F4-6703-01 | 7.41 | 57.88 | -23644 | MALE | Stage IIA | NO | NO | NO | 1456 | Alive |
| TCGA-F4-6704-01 | 62.99 | 139.69 | -21949 | MALE | Stage IIIC | NO | NO | NO | 47 | Alive |
| TCGA-F4-6805-01 | 17 | 50.19 | -21241 | FEMALE | Stage IIA | NO | NO | NO | 1047 | Alive |
| TCGA-F4-6806-01 | 168.47 | 604.39 | -21625 | FEMALE | Stage I | NO | NO | NO | 1260 | Alive |
| TCGA-F4-6807-01 | 65 | 73.63 | -18690 | FEMALE | Stage IIIC | NO | NO | NO | 1309 | Alive |
| TCGA-F4-6808-01 | 139.71 | 636.94 | -19803 | FEMALE | Stage I | NO | NO | NO | 1024 | Alive |
| TCGA-F4-6809-01 | 16.57 | 91.12 | -19039 | FEMALE | Stage IVA | NO | NO |  | 403 | Dead |
| TCGA-F4-6854-01 | 43.21 | 242.33 | -28272 | FEMALE | Stage IIA | NO | NO | NO | 16 | Alive |
| TCGA-F4-6855-01 | 92.66 | 217.41 | -25890 | FEMALE | Stage IIA | NO | NO |  | 1442 | Alive |
| TCGA-F4-6856-01 | 50.59 | 103.92 | -16519 | MALE | Stage I | NO | NO |  | 1074 | Alive |
| TCGA-G4-6293-01 | 28.16 | 127.89 | -18076 | FEMALE | Stage III | NO | NO | NO | 4051 | Alive |
| TCGA-G4-6294-01 | 144.26 | 1521.13 | -27755 | MALE | Stage IV | NO | NO | NO | 858 | Dead |
| TCGA-G4-6295-01 | 41.29 | 157.83 | -25758 | FEMALE | Stage II | NO | NO | NO | 254 | Alive |
| TCGA-G4-6297-01 | 28.53 | 103.53 | -20103 | FEMALE | Stage IV | YES | YES | YES | 2506 | Alive |
| TCGA-G4-6298-01 | 30.52 | 183.36 | -32873 | MALE | Stage IIIB | NO | NO | NO | 715 | Dead |
| TCGA-G4-6299-01 | 158.8 | 553.76 | -25318 | MALE | Stage IIIC | YES | YES | NO | 2268 | Alive |
| TCGA-G4-6302-01 | 1.54 | 3.98 | -32872 | FEMALE | Stage IIA | YES | YES | NO | 2047 | Dead |
| TCGA-G4-6303-01 | 125.54 | 256.66 | -19800 | FEMALE | Stage IV | YES | YES | NO | 2003 | Dead |
| TCGA-G4-6304-01 | 40.31 | 212.92 | -24172 | FEMALE | Stage IIB | NO | NO | NO | 1631 | Alive |
| TCGA-G4-6306-01 | 232.86 | 1154.28 | -26163 | MALE | Stage I |  |  | YES | 1359 | Alive |
| TCGA-G4-6307-01 | 62.24 | 168.9 | -13648 | FEMALE | Stage IIIB | YES | YES |  | 1674 | Alive |
| TCGA-G4-6309-01 | 32.77 | 56.72 | -14729 | FEMALE | Stage IIIB | NO | NO | NO | 2600 | Alive |
| TCGA-G4-6310-01 | 26.7 | 45.37 | -25243 | MALE | Stage IIIB | NO | NO | NO | 1935 | Alive |
| TCGA-G4-6311-01 | 11.79 | 69.77 | -29297 | MALE | Stage III | YES | YES | NO | 1199 | Alive |
| TCGA-G4-6314-01 | 11.54 | 29.93 | -27958 | FEMALE | Stage IV | YES | YES | NO | 1093 | Alive |
| TCGA-G4-6315-01 | 40.51 | 211.04 | -24174 | MALE | Stage IV | NO | NO | NO | 1883 | Alive |
| TCGA-G4-6317-01 | 54.6 | 256.35 | -18822 | FEMALE | Stage IIIC |  |  | NO | 1095 | Alive |
| TCGA-G4-6320-01 | 43.61 | 296.39 | -26840 | MALE | Stage III | YES | YES | NO | 804 | Alive |
| TCGA-G4-6321-01 | 18.84 | 52.49 | -21921 | FEMALE | Stage III | NO | NO | YES | 672 | Alive |
| TCGA-G4-6322-01 | 37.2 | 77.45 | -23825 | MALE | Stage IIIB | YES | YES | NO | 792 | Alive |
| TCGA-G4-6323-01 | 41.9 | 151.74 | -18334 | MALE | Stage IA | NO | NO | NO | 419 | Alive |
| TCGA-G4-6586-01 | 105.05 | 326.71 | -27020 | FEMALE | Stage IIA | YES | YES | NO | 1089 | Alive |
| TCGA-G4-6588-01 | 130.58 | 230.18 | -21532 | FEMALE | Stage IIA | NO | NO | YES | 796 | Alive |
| TCGA-G4-6625-01 | 108.9 | 341.18 | -28163 | FEMALE | Stage IIA | NO | NO | NO | 2792 | Alive |
| TCGA-G4-6626-01 | 249.75 | 1389.73 | -32871 | MALE | Stage IIA | NO | NO | NO | 1422 | Dead |
| TCGA-G4-6627-01 | 10.38 | 50.84 | -30697 | MALE | Stage IIA | NO | NO | NO | 2275 | Alive |
| TCGA-G4-6628-01 | 50.11 | 57.9 | -28772 | MALE | Stage I | NO | NO | NO | 2424 | Alive |
| TCGA-NH-A50T-01 | 70.84 | 277.48 | -25089 | FEMALE | Stage IIA | NO | NO | NO | 553 | Alive |
| TCGA-NH-A50U-01 | 94.3 | 187.71 | -15450 | MALE | Stage IVA | NO | NO |  | 334 | Dead |
| TCGA-NH-A50V-01 | 66.48 | 225.66 | -25467 | MALE | Stage IIIB | NO | NO |  | 588 | Alive |
| TCGA-NH-A5IV-01 | 147.34 | 229.39 | -32872 | FEMALE | Stage IIA | NO | NO | YES | 588 | Alive |
| TCGA-NH-A6GA-01 | 68.04 | 113.9 | -21313 | MALE | Stage IIIC | YES | YES |  | 302 | Dead |
| TCGA-NH-A6GB-01 | 126.67 | 273.4 | -26100 | FEMALE | Stage IIIC | NO | NO |  | 476 | Alive |
| TCGA-NH-A6GC-01 | 54.31 | 172.77 | -24355 | FEMALE | Stage IVB | NO | NO |  | 389 | Alive |
| TCGA-NH-A8F7-01 | 111.53 | 202.23 | -19535 | FEMALE | Stage IIA | NO | NO | NO | 543 | Alive |
| TCGA-NH-A8F8-01 | 51.51 | 97.03 | -29185 | MALE | Stage IV | YES | YES | YES | 511 | Dead |
| TCGA-QG-A5YV-01 | 71.52 | 160.12 | -23499 | FEMALE | Stage IIIC | NO | NO | NO | 1301 | Alive |
| TCGA-QG-A5YW-01 | 70.08 | 69.61 | -20391 | FEMALE | Stage IIIC | NO | NO | NO | 896 | Alive |
| TCGA-QG-A5YX-01 | 37.44 | 96.39 | -22617 | FEMALE | Stage IIA | NO | YES | NO | 1003 | Alive |
| TCGA-QG-A5Z1-01 | 56 | 132.12 | -26220 | MALE | Stage IIIB | YES | YES | NO | 256 | Dead |
| TCGA-QG-A5Z2-01 | 47.07 | 174.13 | -22367 | MALE | Stage I | NO | NO | NO | 952 | Alive |
| TCGA-QL-A97D-01 | 134.56 | 260.51 | -30914 | FEMALE | Stage I |  |  |  | 666 | Alive |
| TCGA-RU-A8FL-01 | 143.46 | 705.9 | -18975 | MALE | Stage IIIB |  |  | NO | 1177 | Alive |
| TCGA-SS-A7HO-01 | 227.88 | 488.18 | -16416 | FEMALE | Stage IIB | YES | YES | NO | 1829 | Alive |
| TCGA-T9-A92H-01 | 144.1 | 376.54 | -30058 | MALE | Stage IIA | NO | NO | NO | 362 | Alive |
| TCGA-WS-AB45-01 | 35.59 | 57.76 |  | FEMALE | Stage IIA | NO | NO | YES | 2130 | Alive |
